# Supplementary material for: Unacylated-Ghrelin Impairs Hippocampal Neurogenesis and Memory in Mice and Is Altered in Parkinson’s Dementia in Humans
Source: Cell Rep Med. 2020 Oct 20;1(7):100120. doi: 10.1016/j.xcrm.2020.100120 (PMC7575905; doi:10.1016/j.xcrm.2020.100120)
Supplement: Document S1. Figures S1–S8 and Table S1 [file mmc1.pdf]

Cell Reports Medicine, Volume *1*

Supplemental Information

Unacylated-Ghrelin Impairs Hippocampal Neurogenesis and Memory in Mice and Is Altered in Parkinson's Dementia in Humans

Amanda K.E. Hornsby, Luke Buntwal, Maria Carla Carisi, Vanessa V. Santos, Fionnuala Johnston, Luke D. Roberts, Martina Sassi, Mathieu Mequinion, Romana Stark, Alex Reichenbach, Sarah H. Lockie, Mario Siervo, Owain Howell, Alwena H. Morgan, Timothy Wells, Zane B. Andrews, David J. Burn, and Jeffrey S. Davies

Figure S1. Hornsby *et al*.

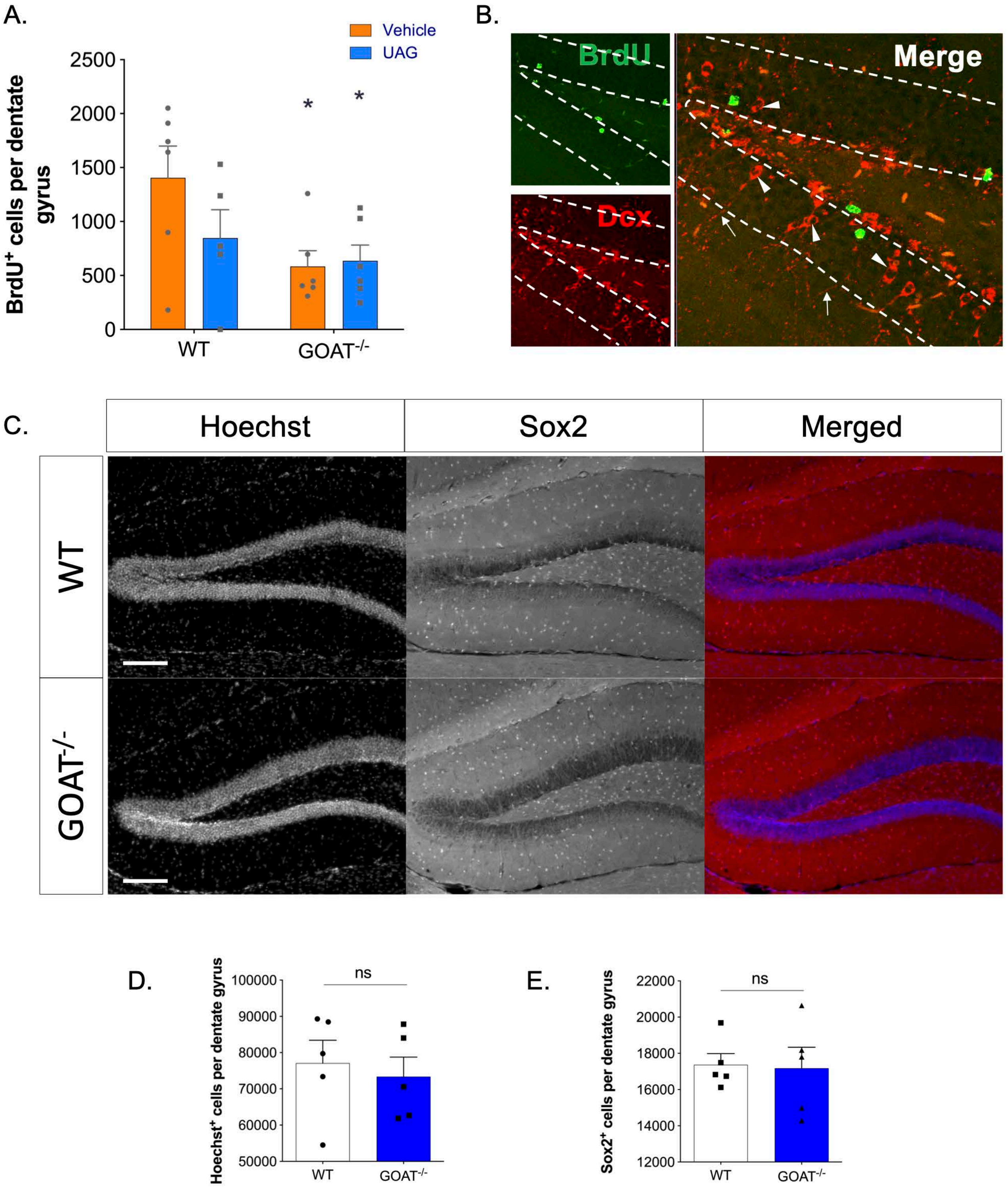

Figure S1. GOAT$^{-/-}$ mice display reduced new BrdU^{+} cell number in the DG compared to WT mice (A). Representative confocal image showing BrdU (green) and Dcx (red) in the wild-type mouse hippocampus. Arrowheads show Dcx^{+} cell bodies within the GCL, whilst arrows depict Dcx^{+} projections extending into the molecular layer (B). Statistical analysis was performed by 2-way ANOVA followed by Holm-Sidak *post-hoc* comparisons vs WT vehicle group (n=5-6 mice/group). Fluorescent images of Hoechst^{+} nuclei and Sox2^{+} NSPCs in DG from WT and GOAT$^{-/-}$ mice (C). GOAT$^{-/-}$ mice do not have gross anatomical changes (Hoechst^{+})(D) or impaired NSPC (Sox2^{+})(E) cell number in the granule cell layer of the DG. Statistical analysis performed by Student's *t*-test (n=5 mice/group). *P<0.05. Scale bar = 200µm. All data shown are mean ± SEM. Related to Figures 1 - 4.

Figure S2. Hornsby *et al.*

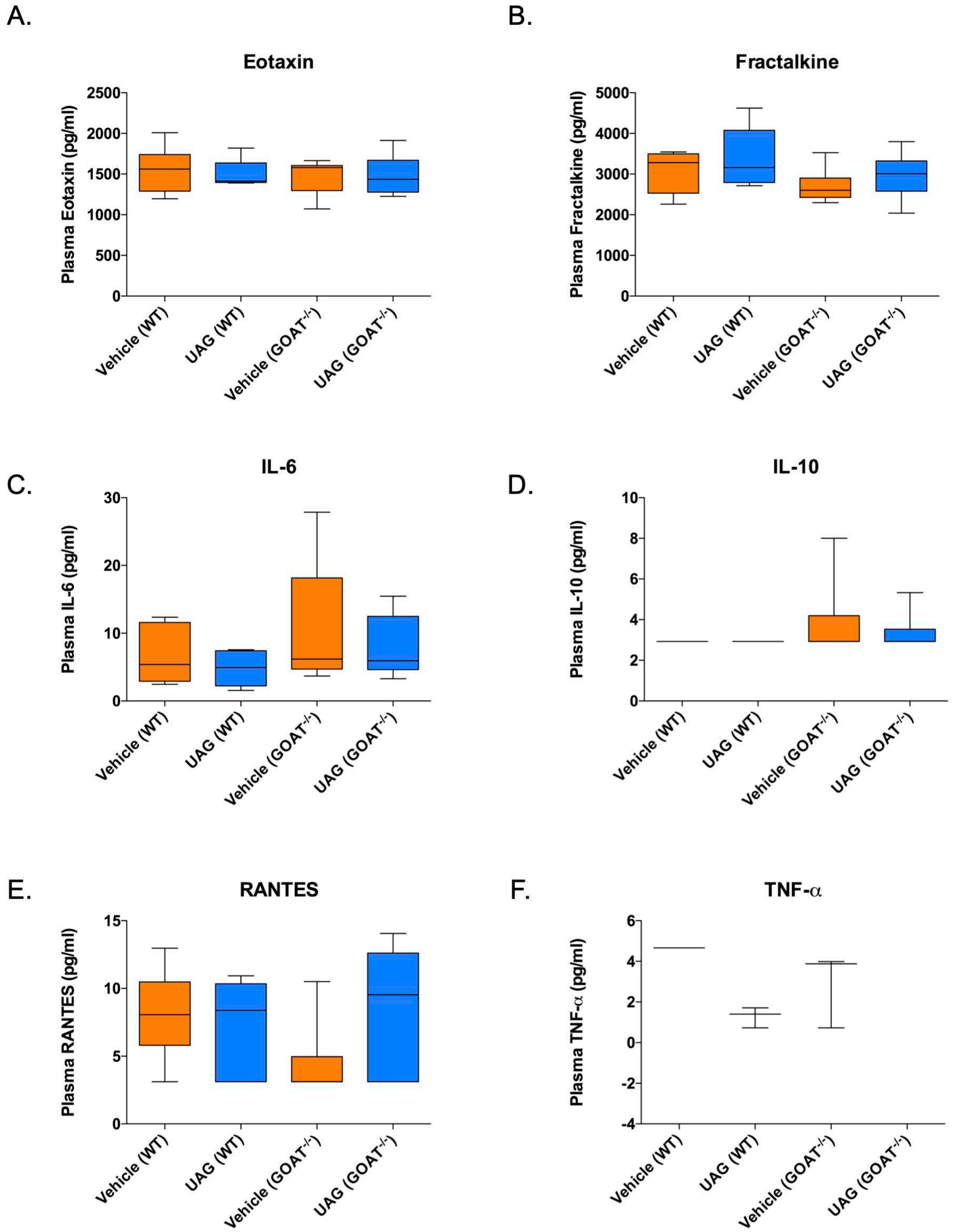

Figure S2. Peripheral administration of unacylated-ghrelin (UAG) or genetic ablation of GOAT does not affect quantities of circulating factors that are known to modulate neurogenesis (A-F). Statistical analysis was performed by 2-way ANOVA followed by Holm-Sidak *post-hoc* comparisons vs WT vehicle group. *P<0.05. *n* = 5-6 mice/group. All data shown are mean ± SEM. Related to Figures 1 – 4.

Figure S3. Hornsby *et al.*

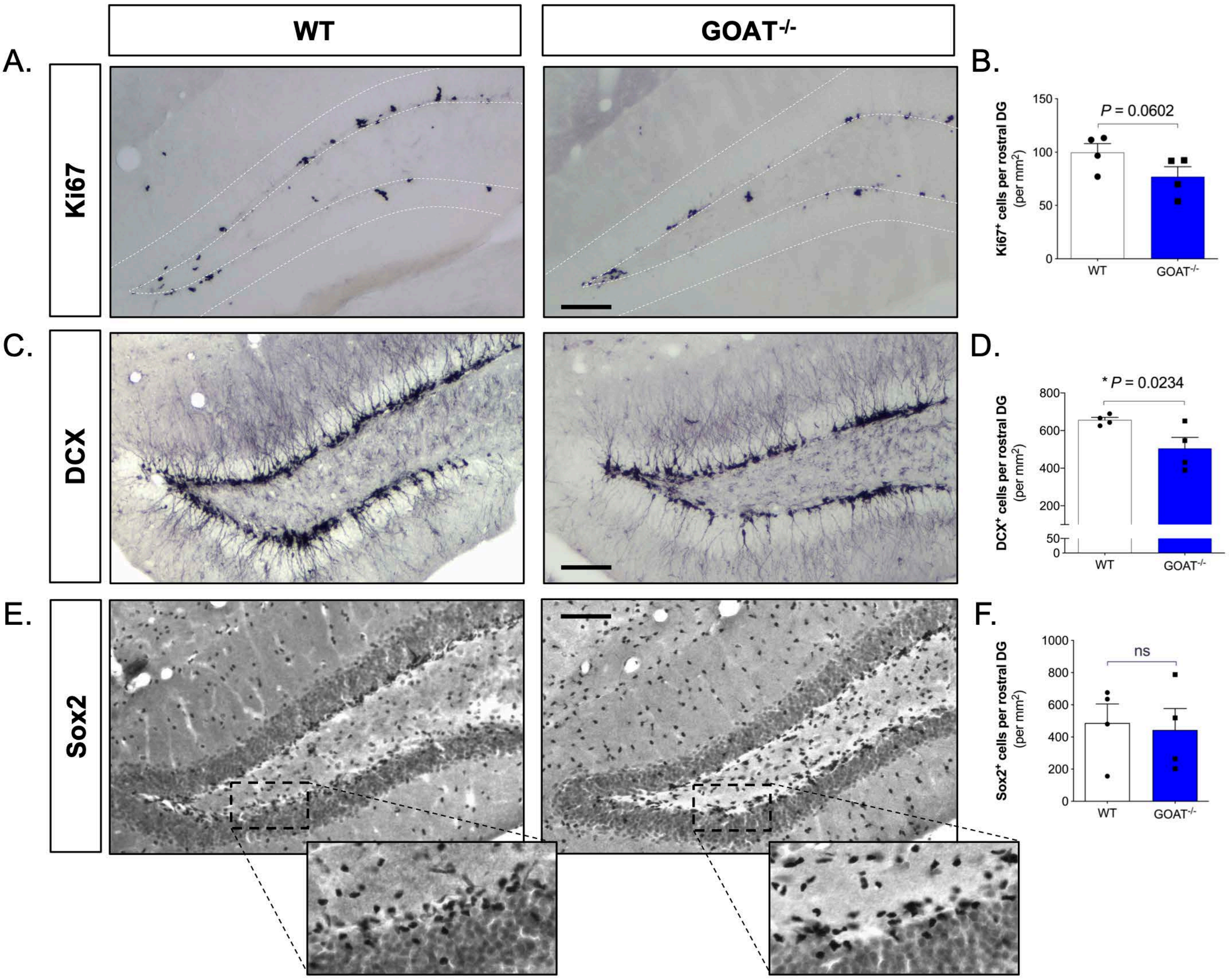

Figure S3. GOAT$^{-/-}$ mice[37] have a reduced rate of cell proliferation (A,B) and neurogenesis (C,D) in the hippocampal DG. However, NSPC (Sox2^{+}) number in the SGZ of the hippocampal DG is similar between WT and GOAT$^{-/-}$ mice (E,F). Brains were collected at 12-weeks of age following intra-cardial perfusion with 4% PFA prior to processing for IHC. Statistical analysis was performed by Student's *t*-test. Scale bar = 200μm. *n* = 4 mice/group. All data shown are mean ± SEM. *P<0.05. Related to Figures 1 and 2.

Figure S4. Hornsby *et al.*

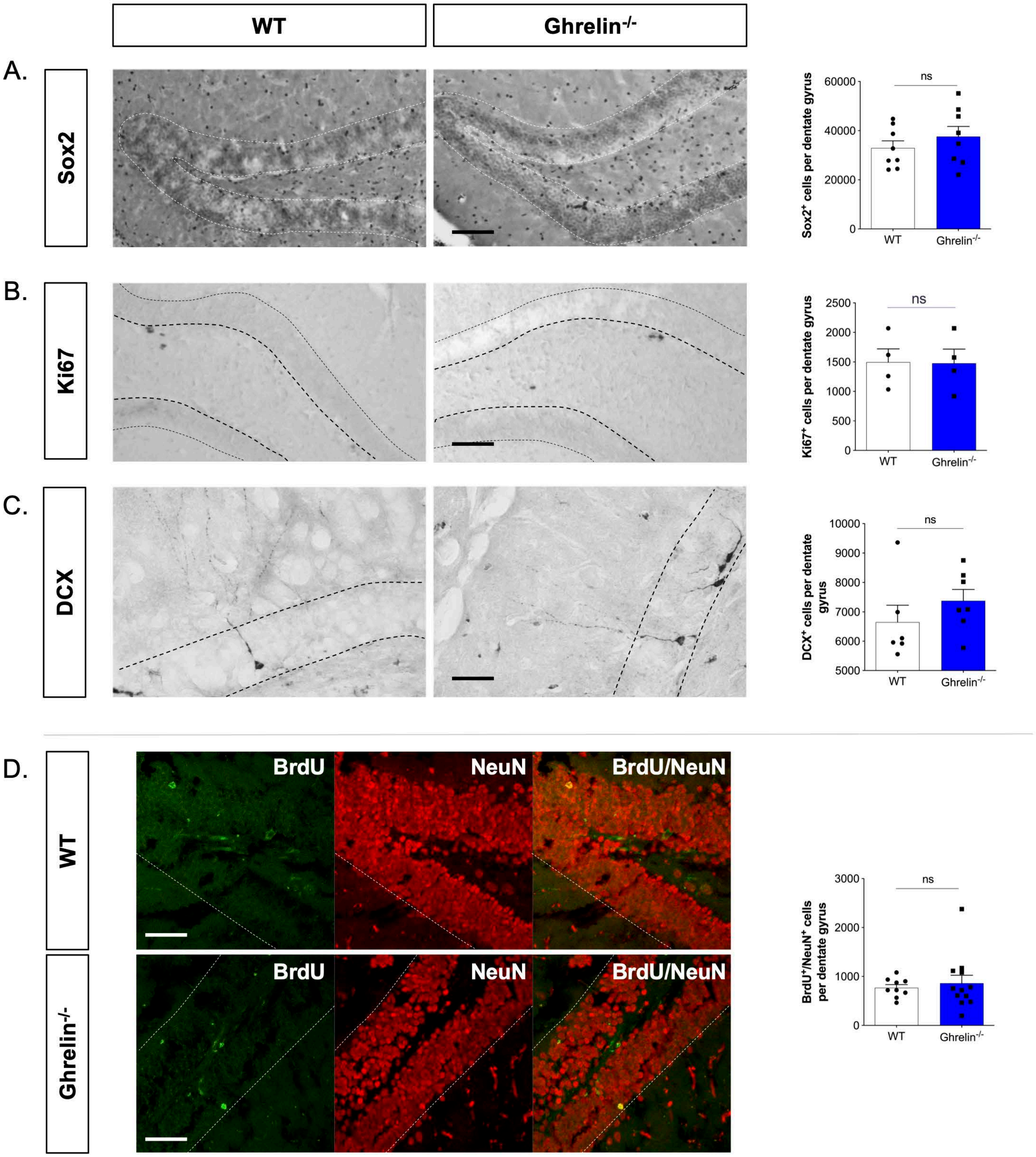

Figure S4. Ghrelin$^{-/-}$ mice have unaltered numbers of Sox2^{+} neural stem cells (*n*=8/group) (A), Ki67^{+} dividing NSPCs (WT *n*=4/group)(B), Dcx^{+} immature neurons (WT *n*=6, ghrelin$^{-/-}$ *n*=7)(C) and BrdU^{+}/NeuN^{+} new adult born neurons (WT *n*=9, ghrelin$^{-/-}$ *n*=12) (D) in the hippocampal DG. Statistical analysis was performed by Students *t*-test. Scale bar = 200μm for A&B, 50μm for C&D. ns = not significant. All data shown are mean ± SEM. Related to Figures 1 and 2.

Figure S5. Hornsby *et al.*

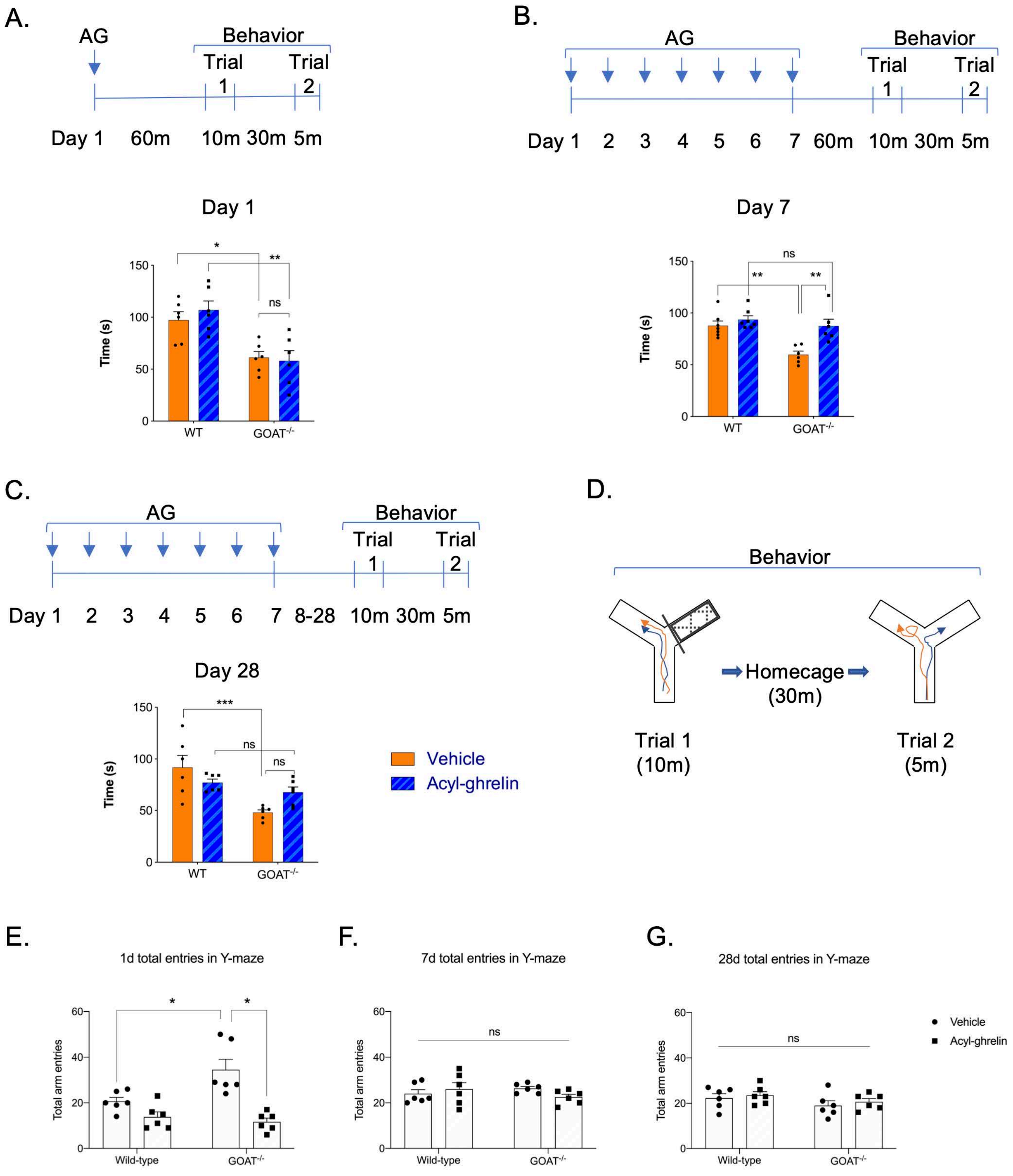

Figure S5. Adult GOAT$^{-/-}$ mice display hippocampal-dependent spatial memory deficits that are rescued by acyl-ghrelin treatment. GOAT$^{-/-}$ mice display memory impairments (A) that are restored by acyl-ghrelin treatment for 7 days (B) and 21 days following the end of treatment on day 28 (C). Schematic representations of treatment timelines (A-C) and behavior using Y-maze (D) are shown. Vehicle-treated GOAT$^{-/-}$ mice display increased total arm entries relative to vehicle-treated wild-type mice that are normalized by acyl-ghrelin treatment on day 1 (E) but show no changes on days 7 and 28 (F,G). Statistical analysis was performed by 2-way ANOVA followed by Holm-Sidak *post-hoc* comparisons (*n*=6 mice/group). *P<0.05, **P<0.01, ***P<0.001. All data shown are mean ± SEM. Related to Figure 4.

Figure S6. Hornsby *et al.*

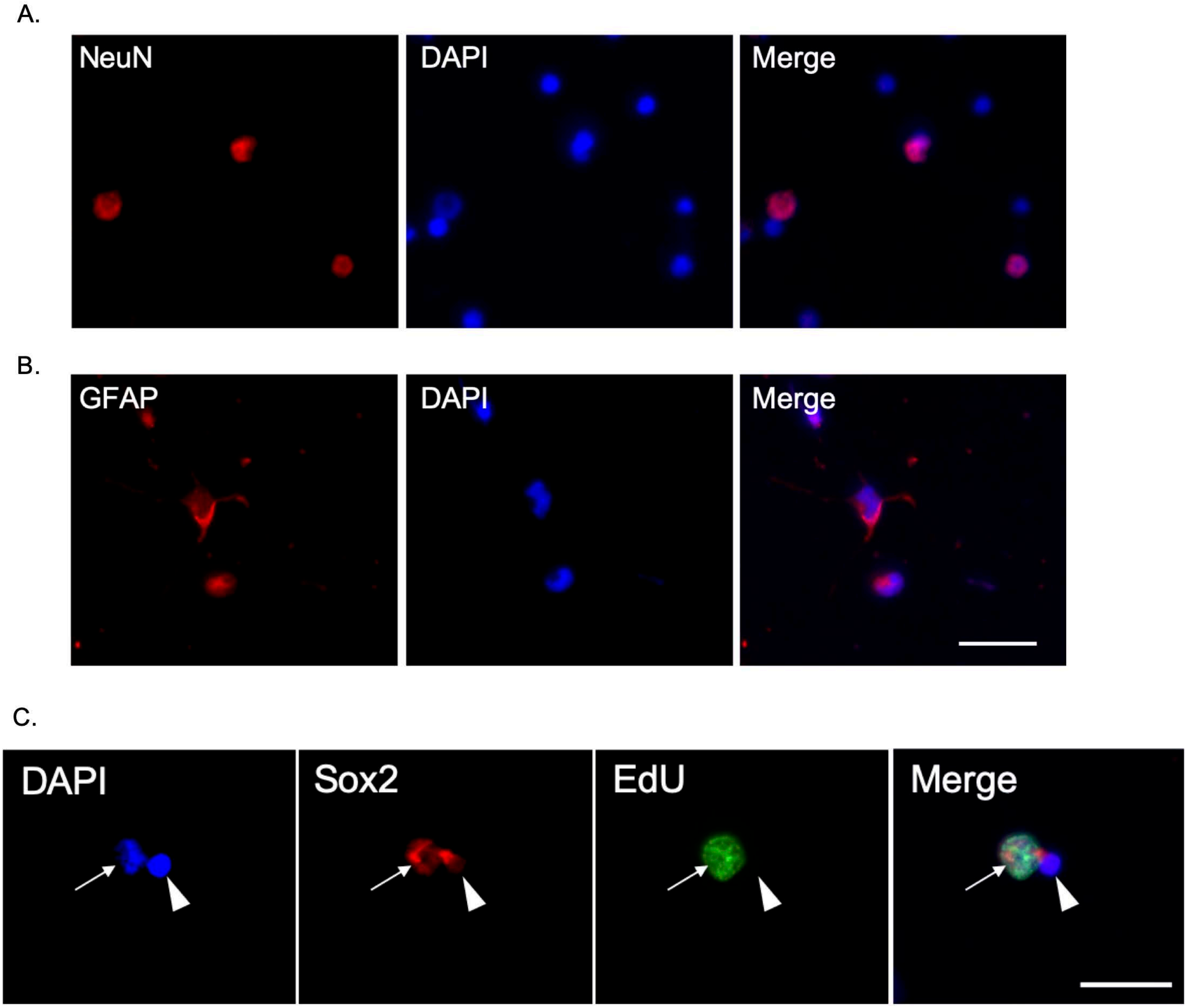

Figure S6. Primary rat hippocampal cell cultures contain a mix of NeuN$^+$ neurons (A) and astrocytes (B) to determine the indirect direct effects of acyl-ghrelin on neurogenesis. These cells also contain dividing (EdU$^+$) NSPCs (Sox2$^+$) identified by the white arrow; whilst a non-dividing NSPC is identified by the white arrow-head (C). Scale bar = 20**μ**m). Related to Figures 5 and 6.

Figure S7. Hornsby *et al.*

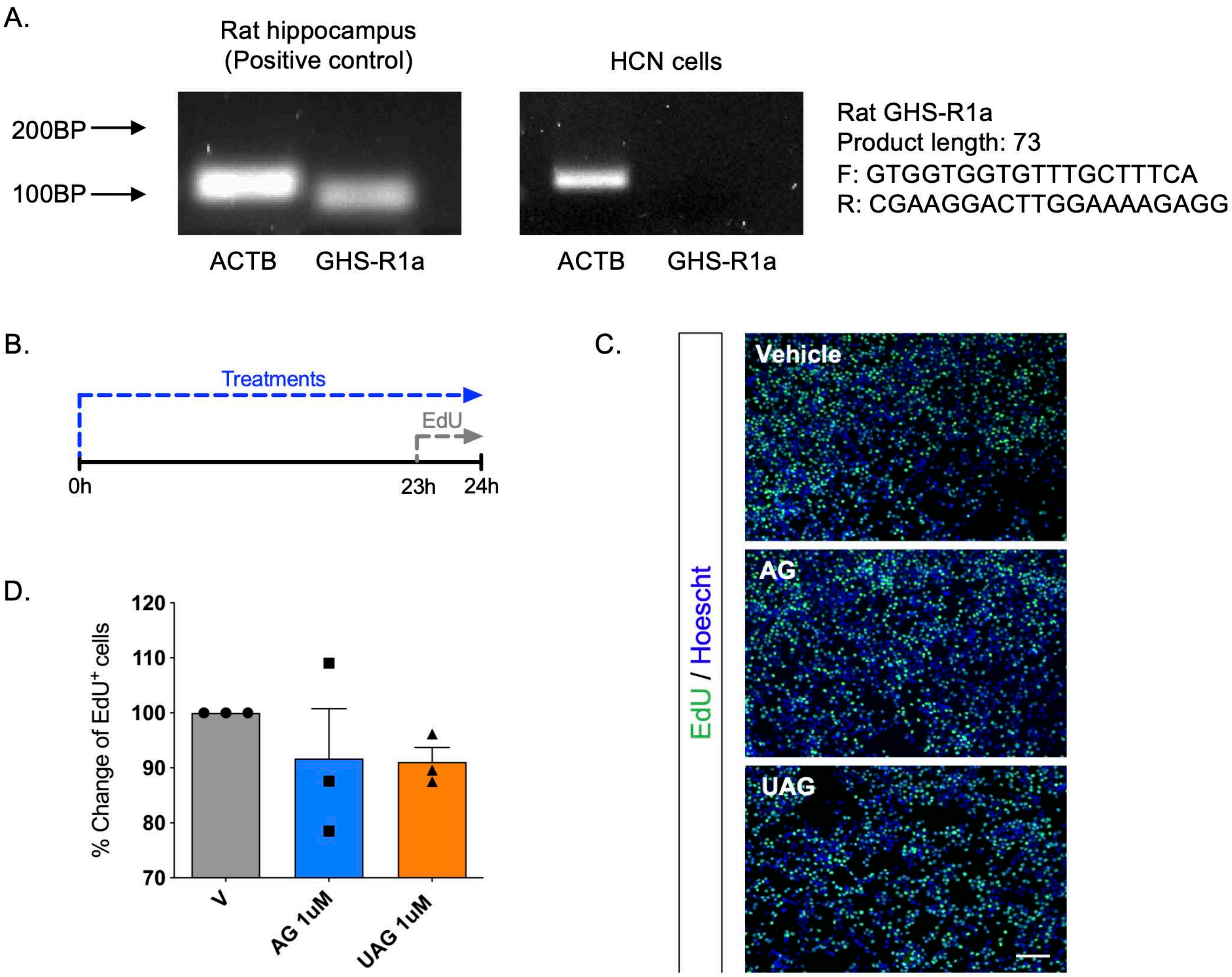

Figure S7. Hippocampal Neural Stem/Progenitor Cells (NSPCs) do not express GHS-R1a. PCR assays reveal that adult rat hippocampal homogenate express GHS-R1a, alongside the housekeeping gene, ACTB. However, rat-derived hippocampal NSPCs (HCN cells), do not express GHS-R1a (A). Schematic of *in-vitro* experiment to determine direct effects on NSPC proliferation (B). Acyl-ghrelin and unacylated-ghrelin do not stimulate cell proliferation (EdU^+) in NSPCs (C, D). Scale bar = 200**μ**m. Related to Figure 6.

Figure S7. Hornsby *et al.*

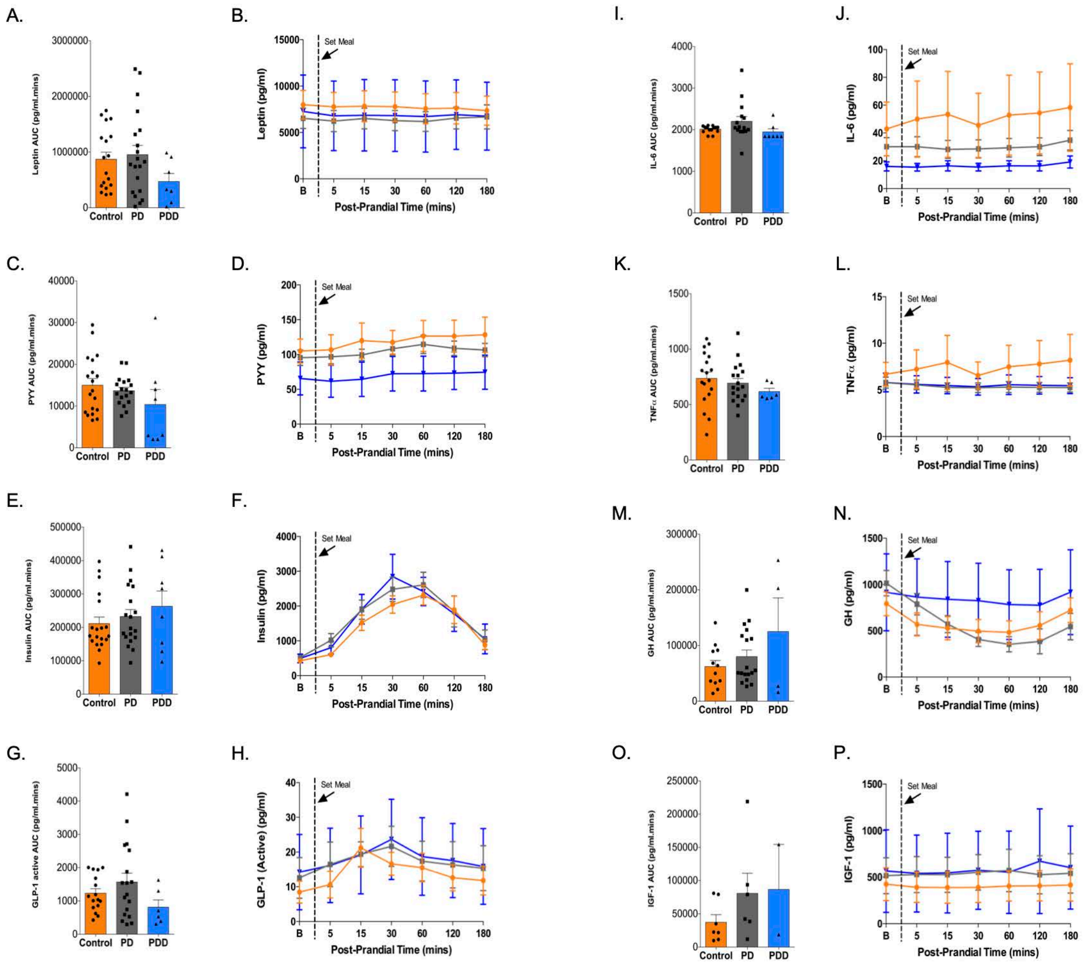

Figure S8. Quantification of circulating factors in healthy, PD and PD dementia subjects. Plasma analysis in healthy controls (n=20), PD (n=20) and PDD (n=8) subjects under fasting and post-prandial conditions. Area under curve (AUC) and plasma values across 180 min demonstrate no change in leptin (A,B), PYY (C,D), insulin (E,F), GLP-1 (active) (G,H), IL-6 (I,J), TNF**α** (K,L), GH (M,N) and IGF-1 (O,P). Dotted line indicates breakfast consumption at time 0. Statistical analysis performed by Kruskal-Wallis test with Dunn's post-hoc multiple comparison revealed no differences between groups. All data shown are mean ± SEM. Related to Figure 7.

Table S1. Hornsby *et al.*

	Healthy controls (*n*=20)	Parkinson's disease (n=20)	Parkinson's disease dementia (*n*=8)	Significance
Age (mean ± sd)	74 ± 6.28	72.2±5.51	74.75 ± 5.99	*P* =0.510
Male (%)	55%	55%	87.5%	*P* =0.229+
MoCA (mean ± sd)	28.35 ± 1.14	27.55 ± 1.47	14.12 ± 5.14	*P* = 0.000*
BMI (mean ± sd)	25.75 ± 2.04	25.31±2.82	24.41 ± 3.20	*P* =0.532
Fasted total ghrelin (pg/ml) (mean ± sem)	541.0±87.98	647.9±108.7	377.8±77.00	*P* =0.2896
Fasted acyl-ghrelin (pg/ml) (mean ± sem)	78.93±13.77	114.4±18.82	51.39±17.24	*P* =0.0925
Post-prandial (180 min) total ghrelin (pg/ml) (mean ± sem)	500.4 ± 65.80	566.1 ± 96.89	401.8 ± 88.61	*P* =0.6464
Post-prandial (180 min) acyl-ghrelin (pg/ml) (mean ± sem)	72.86 ± 10.75	112.0 ± 18.31	41.32 ± 18.25	*P* =0.0169*

Table S1. Demographic information for study participants. MoCA; Montreal Cognitive Assessment. BMI; Body Mass Index. Statistical analysis was performed by Kruskal-Wallis test or the Chi-squared test+. *P <0.05 considered significant. Related to Figure 7.
