# Supplementary material for: Unacylated-Ghrelin Impairs Hippocampal Neurogenesis and Memory in Mice and Is Altered in Parkinson’s Dementia in Humans
Source: Cell Rep Med. 2020 Oct 20;1(7):100120. doi: 10.1016/j.xcrm.2020.100120 (PMC7575905; doi:10.1016/j.xcrm.2020.100120)
Supplement: Document S2. Article plus Supplemental Information [file mmc2.pdf]

Cell Reports
Medicine

Article

Unacylated-Ghrelin Impairs Hippocampal Neurogenesis and Memory in Mice and Is Altered in Parkinson's Dementia in Humans

Graphical Abstract

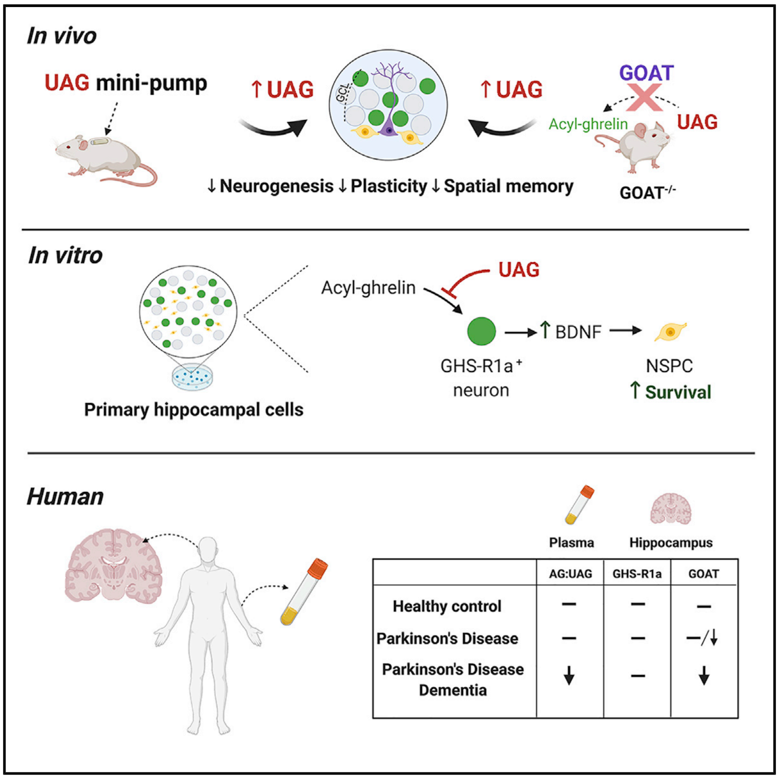

Highlights

- Circulating unacylated-ghrelin (UAG) reduces hippocampal neurogenesis
- Circulating acyl-ghrelin (AG) rescues spatial memory deficit in $GOAT^{-/-}$ mice
- UAG blocks the AG induced survival of newborn hippocampal cells
- Plasma AG:UAG and hippocampal $GOAT^{+}$ cells are reduced in Parkinson's dementia

Authors

Amanda K.E. Hornsby, Luke Buntwal, Maria Carla Carisi, ..., Zane B. Andrews, David J. Burn, Jeffrey S. Davies

Correspondence

jeff.s.davies@swansea.ac.uk

In Brief

The gut-hormone acyl-ghrelin (AG) is known to promote adult hippocampal neurogenesis. Hornsby et al. combine *in vitro* and *in vivo* rodent models, alongside analysis of human plasma, to show that unacylated-ghrelin (UAG) impairs neurogenesis and that the circulating AG:UAG ratio is reduced in Parkinson's dementia.

Hornsby et al., 2020, Cell Reports Medicine *1*, 100120
October 20, 2020 © 2020 The Author(s).
https://doi.org/10.1016/j.xcrm.2020.100120

CellPress

Cell Reports Medicine

CellPress
OPEN ACCESS

Article

Unacylated-Ghrelin Impairs Hippocampal Neurogenesis and Memory in Mice and Is Altered in Parkinson's Dementia in Humans

Amanda K.E. Hornsby,[1,6] Luke Buntwal,[1,6] Maria Carla Carisi,[1] Vanessa V. Santos,[2] Fionnuala Johnston,[3] Luke D. Roberts,[1] Martina Sassi,[1] Mathieu Mequinion,[2] Romana Stark,[2] Alex Reichenbach,[2] Sarah H. Lockie,[2] Mario Siervo,[3,5] Owain Howell,[1] Alwena H. Morgan,[1] Timothy Wells,[4] Zane B. Andrews,[2] David J. Burn,[3] and Jeffrey S. Davies[1,7,*]

[1]Molecular Neurobiology, Institute of Life Sciences, School of Medicine, Swansea University, Swansea, UK
[2]Biomedical Discovery Institute, Department of Physiology, Monash University, Clayton, VIC, Australia
[3]Faculty of Medical Sciences, Newcastle University, Newcastle upon Tyne, UK
[4]School of Biosciences, Cardiff University, Cardiff, UK
[5]School of Life Sciences, Queen's Medical Centre, The University of Nottingham Medical School, Nottingham NG7 2UH, UK
[6]These authors contributed equally
[7]Lead Contact
*Correspondence: jeff.s.davies@swansea.ac.uk
https://doi.org/10.1016/j.xcrm.2020.100120

SUMMARY

Blood-borne factors regulate adult hippocampal neurogenesis and cognition in mammals. We report that elevating circulating unacylated-ghrelin (UAG), using both pharmacological and genetic methods, reduced hippocampal neurogenesis and plasticity in mice. Spatial memory impairments observed in ghrelin-O-acyl transferase-null (GOAT$^{-/-}$) mice that lack acyl-ghrelin (AG) but have high levels of UAG were rescued by acyl-ghrelin. Acyl-ghrelin-mediated neurogenesis *in vitro* was dependent on non-cell-autonomous BDNF signaling that was inhibited by UAG. These findings suggest that post-translational *acylation* of ghrelin is important to neurogenesis and memory in mice. To determine relevance in humans, we analyzed circulating AG:UAG in Parkinson disease (PD) patients diagnosed with dementia (PDD), cognitively intact PD patients, and controls. Notably, plasma AG:UAG was only reduced in PDD. Hippocampal ghrelin-receptor expression remained unchanged; however, GOAT^{+} cell number was reduced in PDD. We identify UAG as a regulator of hippocampal-dependent plasticity and spatial memory and AG:UAG as a putative circulating diagnostic biomarker of dementia.

INTRODUCTION

Circulating factors are known to both enhance[1-4] and impair[5-7] neuronal plasticity and learning in adult mammals. However, the mechanisms underlying these effects are not completely understood. Systemic factors such as Growth Differentiation Factor 11 (GDF11)[1] are reported to regulate the neural stem/progenitor cell (NSPC) niche in the adult hippocampus to promote new neuron formation, termed adult hippocampal neurogenesis (AHN), and cognition. Conversely, circulating Beta-2 microglobulin (B2M)[6] and eotaxin[5] impair the same niche resulting in reduced neurogenesis and impaired cognitive function. These data demonstrate that the hippocampal neurogenic niche is responsive to systemic factors, even in aged mammals, and suggest that circulating factors act as important modulators of mnemonic function.

The birth and maturation of new neurons in the adult hippocampal dentate gyrus (DG) is essential for spatial pattern separation memory,[8,9] which is the ability to separate highly similar components of memories into distinct memory representations.[10] This process is impaired in neurodegeneration[11] and dementia[12] but is enhanced by lifestyle factors, such as exercise[13] and calorie restriction.[14] Indeed, neurogenic impairments observed in the familial Alzheimer disease (FAD) model, 5xFAD, are rescued by exercise. In addition, the suppression of adult neurogenesis was associated with increased neuron loss in 5xFAD, but not wild-type (WT) mice, suggesting a pathophysiological link between impaired AHN and AD progression.[15] Similarly, AHN is impaired in rodent models of Parkinson disease (PD),[16-18] and both NSPC and immature neuron number are reduced in the DG of humans diagnosed with PD dementia (PDD).[11] However, therapeutic strategies that promote AHN are limited.

We recently showed that calorie restriction increased AHN and hippocampal-dependent memory in a mechanism dependent on signaling via the stomach hormone, acyl-ghrelin.[14] Indeed, acyl-ghrelin, which is elevated during calorie restriction, crosses the blood-brain barrier and binds to the growth hormone secretagogue receptor (GHS-R) within the hippocampus and improves spatial memory.[19] Moreover, we showed that peripheral

This is an open access article under the CC BY license (http://creativecommons.org/licenses/by/4.0/).

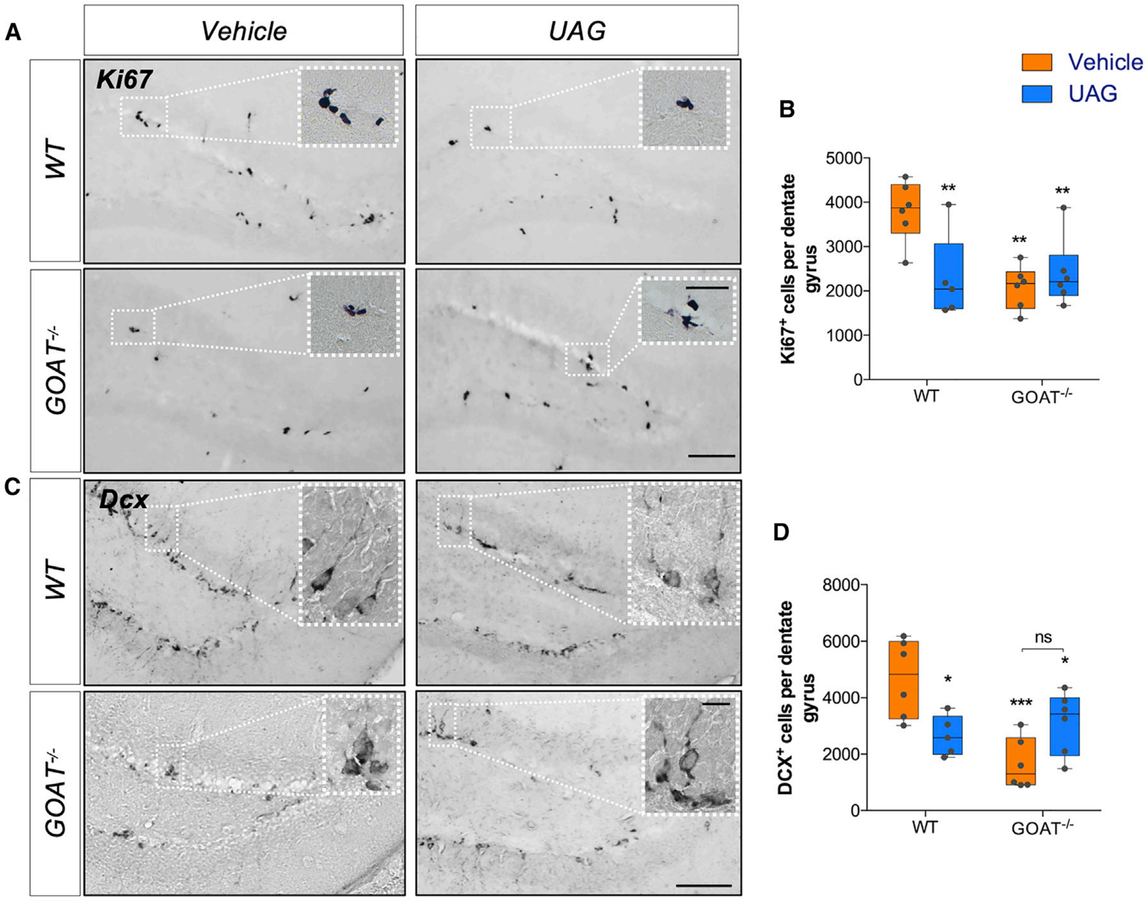

Figure 1. Unacylated-Ghrelin Inhibits Hippocampal Neurogenesis in Adult Mice

Peripheral administration of UAG or genetic ablation of GOAT reduces the number of dividing Ki67+ cells (A and B) and immature Dcx+ neurons (C and D) in the mouse DG. Statistical analysis was performed by 2-way ANOVA followed by Holm-Sidak post hoc comparisons. Scale bar, 200 μm (inset scale bar, 20 μm). *$p < 0.05$, **$p < 0.01$, ***$p < 0.001$ versus WT vehicle group. Data shown are mean ± SEM n = 5–6 mice/group.

injection of acyl-ghrelin, at physiological doses, increases AHN and enhances pattern separation memory in adult rats.[20] Similarly, deletion of GHS-R in mice results in increased susceptibility to chronic stress coupled with reduced neurogenesis in the ventral DG.[21] These studies clearly identify acyl-ghrelin as an important pro-neurogenic circulating factor.[22] In order to generate acyl-ghrelin, ghrelin must undergo post-translational acylation by the enzyme ghrelin-O-acyl transferase (GOAT),[23,24] prior to binding and activating GHS-R signaling.[25] Unacylated-ghrelin (UAG) represents ~80%–90% of circulating ghrelin and is often considered an inactive precursor to acyl-ghrelin. However, there is growing evidence that UAG functions as a hormone distinct from acyl-ghrelin and GHS-R. For example, UAG induces genome-wide changes in the expression of genes linked to glucose and lipid metabolism in fat, muscle, and liver from GHS-R$^{-/-}$ mice,[26] providing evidence for the existence of a UAG receptor that is yet to be identified. UAG also inhibits acyl-ghrelin actions that are mediated by GHS-R.[27-29] For example, UAG suppressed acyl-ghrelin-induced neuronal activity in the brainstem and prevented the acyl-ghrelin/GHS-R-mediated increase in food intake.[30] We therefore sought to determine whether UAG modulates hippocampal plasticity and memory function and whether plasma levels of acyl-ghrelin and UAG associate with dementia in humans.

RESULTS

UAG Inhibits Hippocampal Neurogenesis in Adult Mice

To assess whether UAG regulates adult NSPC plasticity in the sub-granular zone (SGZ) of the hippocampus, we analyzed the effect of UAG administered peripherally for 7 days in WT and GOAT$^{-/-}$ mice.[23] GOAT$^{-/-}$ mice lack circulating acyl-ghrelin but have elevated levels of UAG making them ideally suited to assessing the loss of acyl-ghrelin coupled with increased plasma UAG.[31] Surprisingly, UAG-treated WT mice showed a significant 40% decrease in Ki67+-proliferating cells in the SGZ compared to vehicle-treated WT mice ($p = 0.0056$) (Figures 1A and 1B). Similarly, genetic blockade of acyl-ghrelin signaling in GOAT$^{-/-}$ mice reduced the number of dividing Ki67+ progenitor cells in the SGZ ($p = 0.0020$) (main effect of genotype, $p = 0.0175$, $F(1,19) = 6.766$; interaction between genotype and treatment, $p = 0.0074$, $F(1,19) = 9.003$) (Figures 1A and 1B). These findings were

CellPress
OPEN ACCESS

accompanied by a significant reduction in the number of doublecortin positive (Dcx^+) immature neurons within the SGZ of both UAG-treated WT (p = 0.0106) and vehicle-treated $GOAT^{-/-}$ mice (p = 0.0003) (main effect of genotype, p = 0.0096, $F(1,19) = 8.287$; interaction between genotype x treatment, p = 0.0009, $F(1,19) = 15.33$) (Figures 1C and 1D). UAG did not further exacerbate the reduction in proliferating cells and immature neurons in $GOAT^{-/-}$ mice, suggesting that UAG's effect on neurogenesis may be via a saturable mechanism. The responsiveness of the $GOAT^{-/-}$ neurogenic niche to UAG treatment may be attenuated by developmental compensation or exposure to consistently high levels of UAG throughout development. While UAG is often considered inactive, reports of UAG opposing acyl-ghrelin function on hepatocyte gluconeogenesis,[32] blocking acyl-ghrelin-induced food intake[33] and suppressing activity of the GH axis,[34] provide support for our findings. Together, these data suggest that the striking decrease in hippocampal cell proliferation and immature neuron number is due to elevated UAG, rather than simply loss of acyl-ghrelin.

UAG Reduces the Number of New Adult-Born Immature Neurons and Non-neuronal Cells in the Adult Hippocampus

Next, as ~70% of young adult-born neurons undergo *Bax*-mediated programmed cell death during an early stage of differentiation,[35] we studied whether UAG reduced the number of new adult-born DG neurons. To achieve this, mice in the above study received an injection of BrdU to birth-date-proliferating cells on day 2 of the infusion prior to brain dissection and immunochemical analysis on day 7. The number of immature neurons ($BrdU^+/Dcx^+$) was significantly reduced in $GOAT^{-/-}$ mice[36] (main effect of genotype, p = 0.0171, $F(1,18) = 6.896$) (Figures 2A and 2D; Figure S1B). There was also a ~30% reduction in the mean number of $BrdU^+/Dcx^+$ cells in UAG-treated WT mice; however, this was not statistically significant (p = 0.2757) (Figure 2A). Further analysis revealed a significant reduction in the number of new adult-born cells that lack Dcx immunoreactivity ($BrdU^+/Dcx^-$) in UAG-treated WT mice, relative to vehicle-treated WT mice (p = 0.0288) (interaction between genotype x treatment, p = 0.022, $F(1,18) = 6.282$) (Figure 2B). Indeed, quantification of the relative proportion of new adult-born cell types demonstrated that UAG reduced the proportion of $BrdU^+/Dcx^-$ cells in WT mice (p = 0.0029) (main effect on cell type, p = 0.0033, $F(1, 36) = 9.924$; interaction between treatment x cell type, p = 0.0276, $F(3, 36) = 3.413$) (Figure 2C). These data suggest that UAG may play an important role in regulating the number of new astro-glial cells or new stem cells originating following asymmetric cell division.

Analysis of a second mouse model with genetic deletion of GOAT[37] revealed a similar reduction in DG neurogenesis (Figures S3A–S3D). Importantly, genetic deletion of GOAT, in either knockout model, did not affect the number of type II stem cells ($Sox2^+$) in the SGZ (p = 0.4433, Figures S1C and S1E and p = 0.4075 Figures S3E and S3F). To avoid counting a subset of astrocytes that co-express Sox2, we performed double immunofluorescence, using anti-Sox2 and the astrocyte-specific anti-S100β antibodies. Our findings demonstrate unaltered expression of $Sox2^+/S100\beta^-$ cells in the SGZ of $GOAT^{-/-}$ mice (3,012 ± 114.0 cells) relative to WT mice (3,001 ± 133.8 cells) (p = 0.9964) (Figures 2E and 2F), suggesting that the observed reduction in dividing progenitors was not due to a reduced stem cell pool but by modulation of the adult neurogenic niche. To determine whether circulating immune factors, that are known to inhibit AHN, were altered in UAG- and vehicle-treated $GOAT^{-/-}$ and WT mice, we quantified plasma levels of eotaxin, fractalkine, interleukin-6 (IL-6), IL-10, RANTES, and tumor necrosis factor-α (TNF-α). However, there were no significant alterations in levels of these circulating factors in $GOAT^{-/-}$ mice or in WT mice following UAG treatment (Figure S2). These data identify GOAT-mediated acylation of ghrelin as an important modulator of AHN, with UAG providing anti-neurogenic activity opposing acyl-ghrelin's pro-neurogenic effects.[14,20]

To further test this finding, we quantified neurogenesis in mice with genetic ablation of the ghrelin gene. These $ghrelin^{-/-}$ mice[38], which lack both acyl-ghrelin and UAG, had no impairments in adult neurogenesis. A previous study reported that the rate of cell proliferation and neuronal differentiation were reduced in 8- to 9-week-old male $ghrelin^{-/-}$ mice compared to non-littermate controls.[39] However, our analyses of total NSPC number (Sox2; p = 0.3674), cell proliferation (Ki67; p = 0.4797), immature neuron number (Dcx; p = 0.1527), and new adult-born neuron number (BrdU/NeuN; p = 0.6409) throughout the rostro-caudal extent of the hippocampus in 6-month-old male and female $ghrelin^{-/-}$ mice confirmed no change in any of these measures relative to WT, sex-matched, littermate control mice (Figure S4). Our data from $ghrelin^{-/-}$ mice are consistent with the idea that the loss of pro-neurogenic acyl-ghrelin and anti-neurogenic UAG resulted in no net change in AHN. In contrast, $GOAT^{-/-}$ mice lack acyl-ghrelin but have high levels of UAG, suggesting that high circulating UAG is at least partly responsible for the reduction in AHN, rather than simply the loss of acyl-ghrelin. Indeed, this interpretation is consistent with our data showing that elevated UAG in WT mice results in reduced hippocampal plasticity.

UAG and $GOAT^{-/-}$ Reduce Markers of Hippocampal Plasticity in Adult Mice

Next, we assessed whether elevation of UAG disrupted other molecular signatures of hippocampal impairment. The immediate early gene (IEG), c-Fos, which is associated with changes in neuronal gene expression that promote learning and memory function,[40,41] was quantified in the DG of vehicle- or UAG-treated WT and $GOAT^{-/-}$ mice. In keeping with our findings of impaired plasticity, we observed a significant reduction in immuno-labeling of c-Fos in both UAG-treated WT (58%, p = 0.0147) and $GOAT^{-/-}$ (44%, p = 0.0377) mice (main effect of treatment, p = 0.0392, $F(1,16) = 5.046$; interaction between genotype x treatment, p = 0.0343, $F(1,16) = 5.354$) (Figures 3A and 3B). In addition, as acyl-ghrelin promotes F-actin expression in hippocampal dendrites it is linked with the generation and re-modeling of spines.[42] Therefore, we analyzed dendritic spines on hippocampal neurons from WT and $GOAT^{-/-}$ mice. While there was no change in total spine number between the two groups (p = 0.4695, Figure 3C), our analysis revealed a significant 53% reduction in immature "stubby" spine number in $GOAT^{-/-}$ mice (p = 0.0164) (main effect of spine type, p = 0.0001, $F(4,20) = 26.36$) (Figures 3D and 3E). Dendritic spines form excitatory synapses with pre-synaptic axons and are essential for

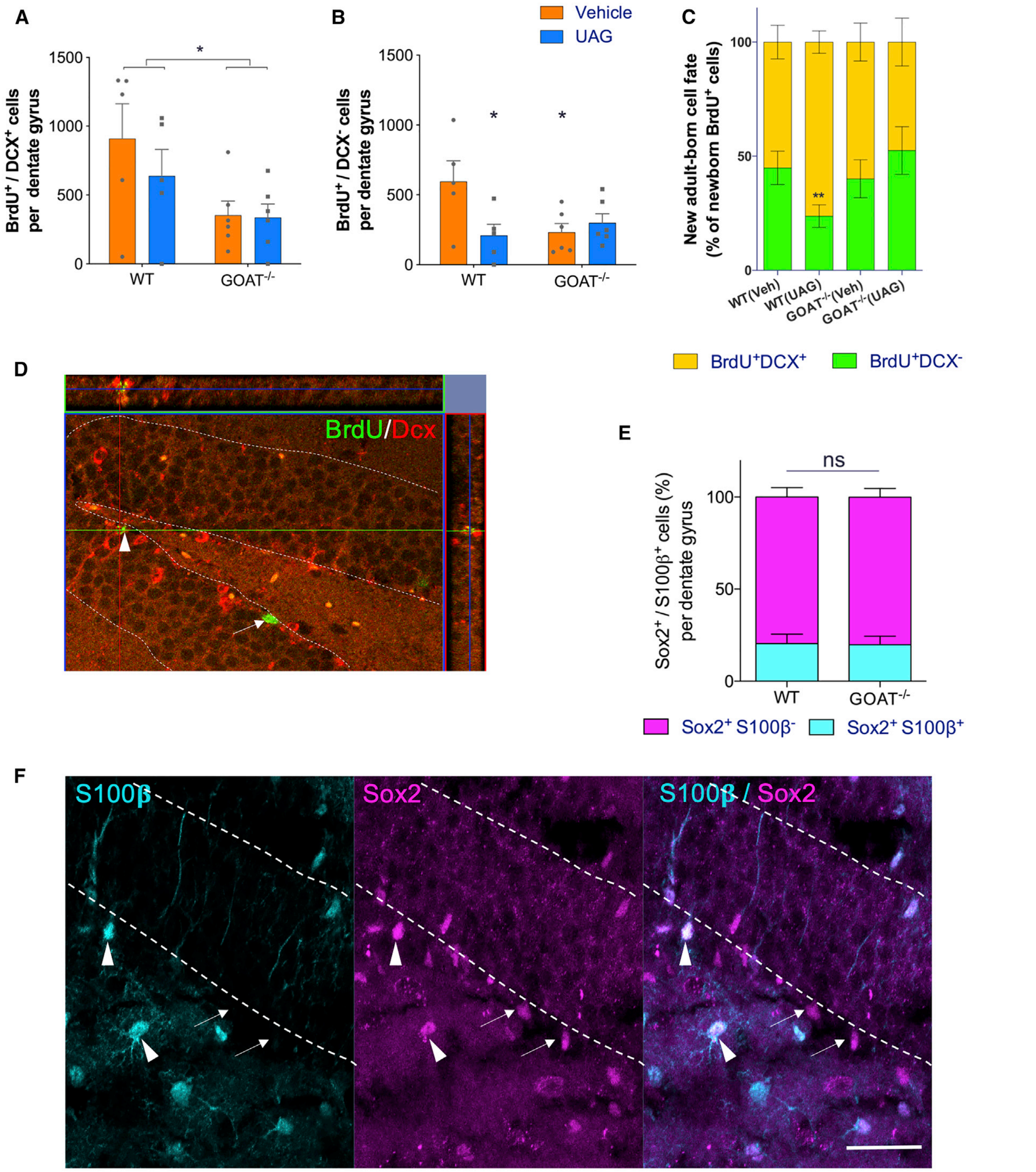

Figure 2. Unacylated-Ghrelin Reduces the Number of New Adult-Born Non-neuronal Cells

(A–C) Genetic ablation of GOAT reduced the number of new neuroblasts (main effect of genotype) (A) and new non-neuronal cells (B). UAG treatment reduced the number of new non-neuronal cells (B, C).

(D) Representative confocal image of DG from wild-type mouse showing new $BrdU^+/Dcx^+$ neuroblast (arrowhead) and a new $BrdU^+/Dcx^-$ non-neuronal cell (arrow), n = 5–6 mice/group.

(E) Type II NSPC number was unaltered in the DG of $GOAT^{-/-}$ mice, n = 3 mice/group.

(F) Representative confocal image of DG from wild-type mouse showing $Sox2^+/S100\beta^-$ type II NSPCs (arrow) and $Sox2^+/S100\beta^+$ astrocytes (arrowhead) in the DG.

Statistical analysis was performed by 2-way ANOVA followed by Holm-Sidak post hoc comparisons. Scale bar, 50 μm. $^*p < 0.05$, $^{**}p < 0.01$, versus WT vehicle group. Data shown are mean ± SEM.

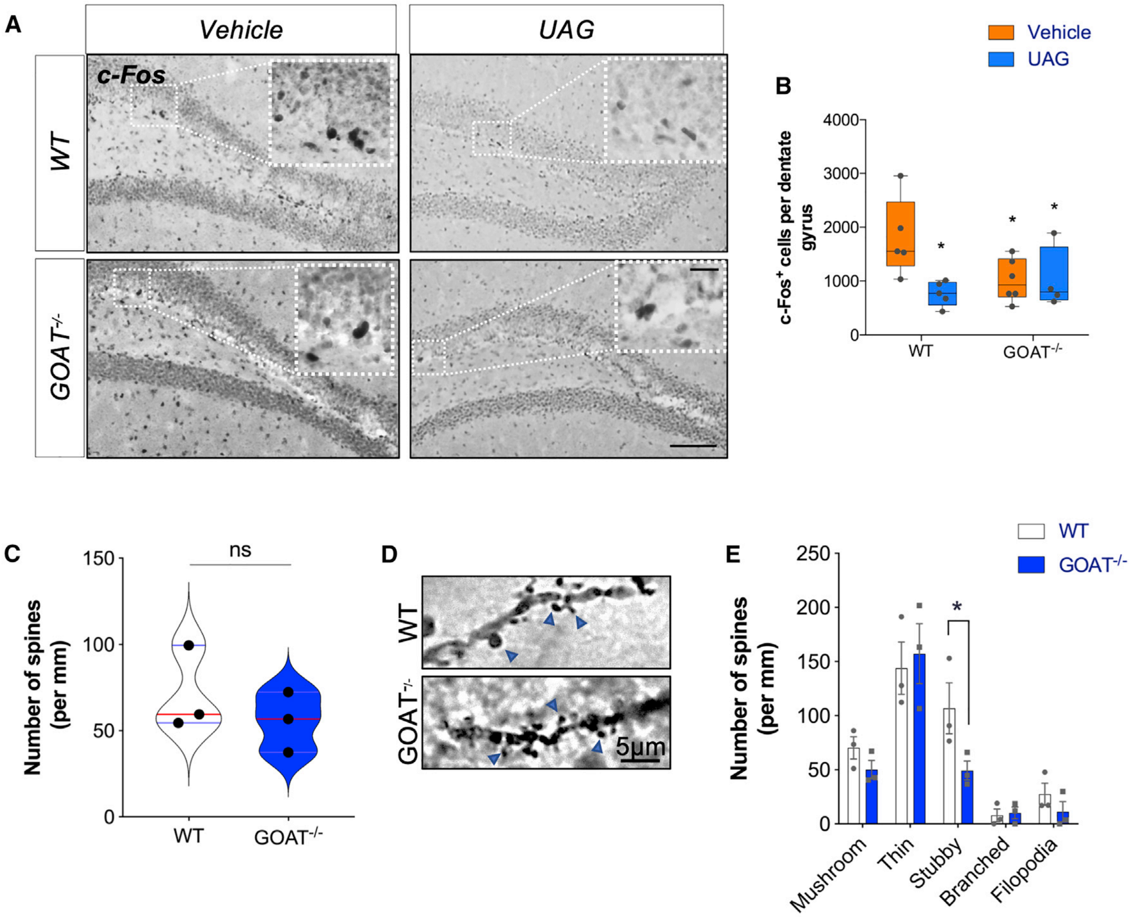

Figure 3. UAG and GOAT$^{-/-}$ Reduce Hippocampal Plasticity in Adult Mice

(A and B) Peripheral administration of UAG or genetic ablation of GOAT reduces the number of c-Fos^{+} neurons in the mouse DG. Statistical analysis was performed by 2-way ANOVA followed by Holm-Sidak post hoc comparisons. Scale bar, 200 μm (inset scale bar, 20 μm). *p < 0.05 versus WT vehicle group. Data shown are mean ± SEM n = 5–6 mice/group.

(C–E) Hippocampal dendritic spine number was unaltered in GOAT$^{-/-}$ mice (C and D); however, spine sub-type analysis demonstrated a significant reduction in the number of stubby spines (E). For spine analysis, statistical analysis was performed by Student's t test (C) and two-way ANOVA followed by Fisher's LSD test (E). Scale bar, 5 μm. n = 3 mice/group. All data shown are mean ± SEM. *p < 0.05 versus WT group.

synaptic plasticity, with spine morphology linked to cognitive function.[43] Given the established role of hippocampal c-Fos[27-29] and dendritic spines[44] in regulating plasticity and cognition, we reasoned that hippocampal-dependent learning and memory may be impaired in GOAT$^{-/-}$ mice.

Adult GOAT$^{-/-}$ Mice Display Hippocampal-Dependent Spatial Memory Deficits that Are Rescued by Acyl-ghrelin Treatment

To determine whether the neurochemical and structural deficits in hippocampal plasticity-related factors observed in GOAT$^{-/-}$ mice resulted in memory impairments, adult WT and GOAT$^{-/-}$ mice were tested using the hippocampal-dependent spatial memory Y-maze task (Figure 4). GOAT$^{-/-}$ mice displayed a deficit in performance compared to WT mice, entering the novel arm fewer times ($p = 0.0023$; interaction between arm choice x genotype, $p = 0.0007$, $F(6, 60) = 4.607$. Figure 4A) and spending significantly less time in the novel arm of the Y-maze ($p = 0.0168$; main effect of genotype, $p = 0.0001$, $F(1,20) = 26.70$. Figure S5A) compared to WT mice. To assess whether this deficit could be rescued by acyl-ghrelin, WT and GOAT$^{-/-}$ mice were given daily injections of either saline or acyl-ghrelin for one or seven days prior to Y-maze testing. Acyl-ghrelin treatment 1 h before testing did not alter performance in GOAT$^{-/-}$ mice relative to vehicle-treated GOAT$^{-/-}$ mice (entries into novel arm, $p = 0.1692$ (Figure 4A); time in novel arm, $p = 0.7972$ (Figure S5A). However, treatment with acyl-ghrelin for 7 days enhanced performance relative to vehicle-treated GOAT$^{-/-}$ mice (entries into novel arm, $p = 0.0114$ (Figure 4B); time in novel arm, $p = 0.0019$ [Figure S5B]) and rescued the deficit in GOAT$^{-/-}$ mice relative to acyl-ghrelin-treated WT mice (entries into novel arm, $p = 0.8263$ [Figure 4B]; time in novel arm, $p = 0.7254$ [Figure S5B]); main effect of arm choice, $p \leq 0.0001$, $F(2, 60) = 145.1$; interaction between arm choice x genotype, $p \leq 0.0001$, $F(6, 60) = 7.426$ [Figure 4B]). Interestingly, the rescue of spatial memory performance in acyl-ghrelin-treated GOAT$^{-/-}$ mice

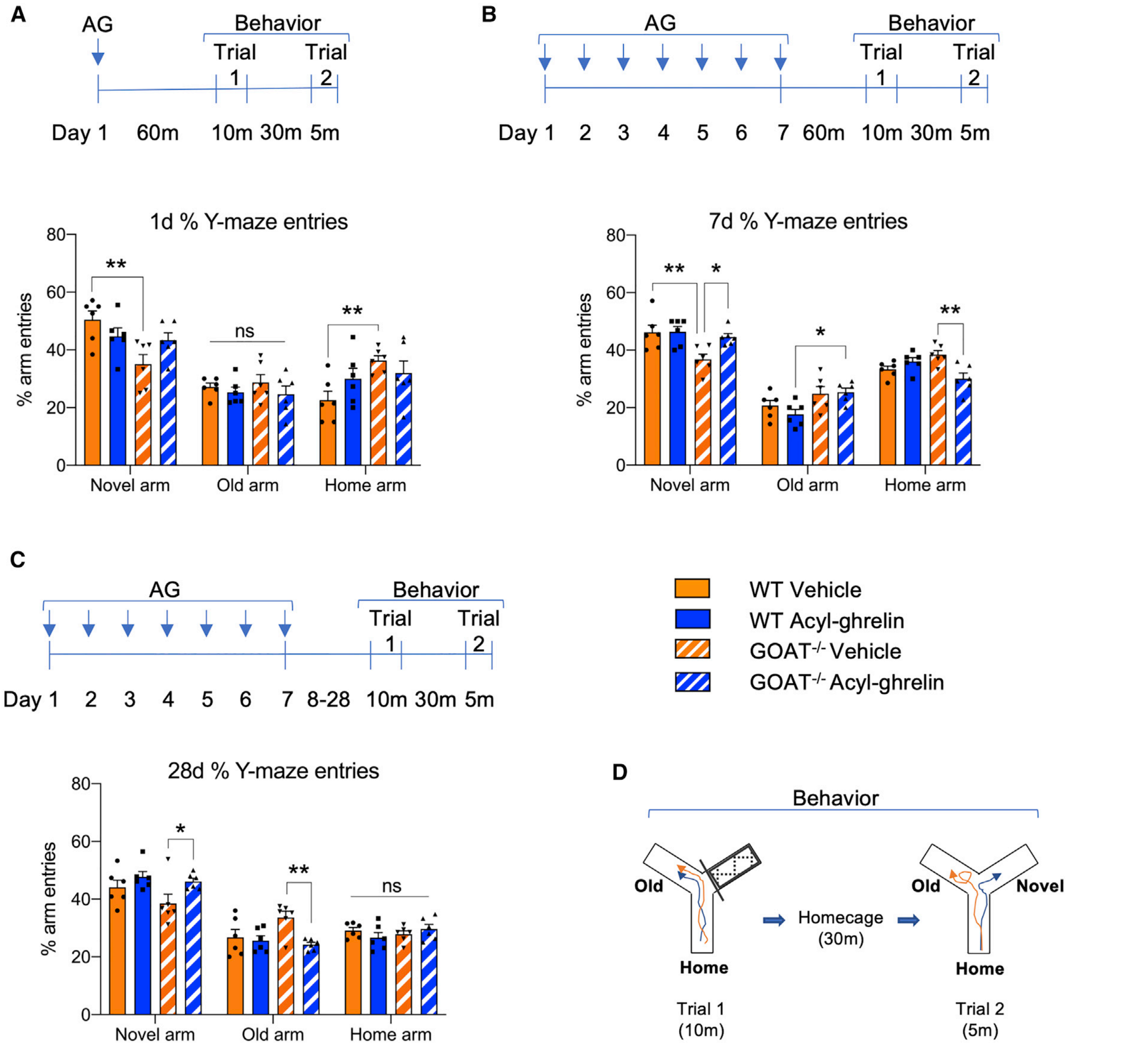

Figure 4. Adult GOAT$^{-/-}$ Mice Display Hippocampal-Dependent Spatial Memory Deficits that Are Rescued by Acyl-ghrelin Treatment

(A–C) Analysis of relative entries into each arm of the Y-maze shows that GOAT$^{-/-}$ mice enter the novel arms less often (A and B; $p < 0.01$). Acyl-ghrelin treatment for 7 days significantly increases the number of entries to the novel arm in GOAT$^{-/-}$ mice on day 7 (B, $p < 0.05$) and day 28 (C, $p < 0.05$).

(D) Schematic representations of Y-maze apparatus, indicating novel, old, and home arms.

Statistical analysis was performed by 2-way ANOVA followed by Holm-Sidak post hoc comparisons (n = 6 mice/group). *$p < 0.05$, **$p < 0.01$. All data shown are mean ± SEM.

relative to vehicle-treated GOAT$^{-/-}$ mice was observed on day 28, 21 days following the end of treatment (entries into novel arm, p = 0.0357 [Figure 4C]). Similarly, acyl-ghrelin-treated WT mice and GOAT$^{-/-}$ mice performed comparably at this time point (entries into novel arm, p = 0.7069 [Figure 4C]; time in novel arm, p = 0.3413 [Figure S5C]; main effect of arm choice, $p \leq 0.0001$, $F(2, 60) = 92.78$; interaction between arm choice x genotype, p = 0.0006, $F(3, 60) = 4.696$ [Figure 4C]). Analysis of total Y-maze arm entries suggest that GOAT$^{-/-}$ mice do not have deficits in exploration (Figures S5E–S5G). These data are consistent with our neurochemical findings and demonstrate that GOAT is essential for intact hippocampal-dependent spatial memory.

UAG Inhibits Acyl-Ghrelin-Mediated Hippocampal Cell Proliferation and New Cell Survival *In Vitro*

To determine whether the ghrelin peptides mediate direct effects on the hippocampus, we used the thymidine analog, EdU, to quantify cell proliferation and survival following treatment of hippocampal cell cultures with acyl-ghrelin or UAG. The primary hippocampal culture system, containing a mix of cell types (Figure S6), was used to show that 24 h acyl-ghrelin treatment significantly increased, in a dose dependent-manner, the number of dividing EdU^{+} cells labeled in the final hour of treatment (p = 0.0186 [100 nM], p = 0.0205 [1,000 nM]). Similar treatment with UAG had no effect (p = 0.9814 [100 nM], p = 0.8033 [1,000 nM]

CellPress
OPEN ACCESS

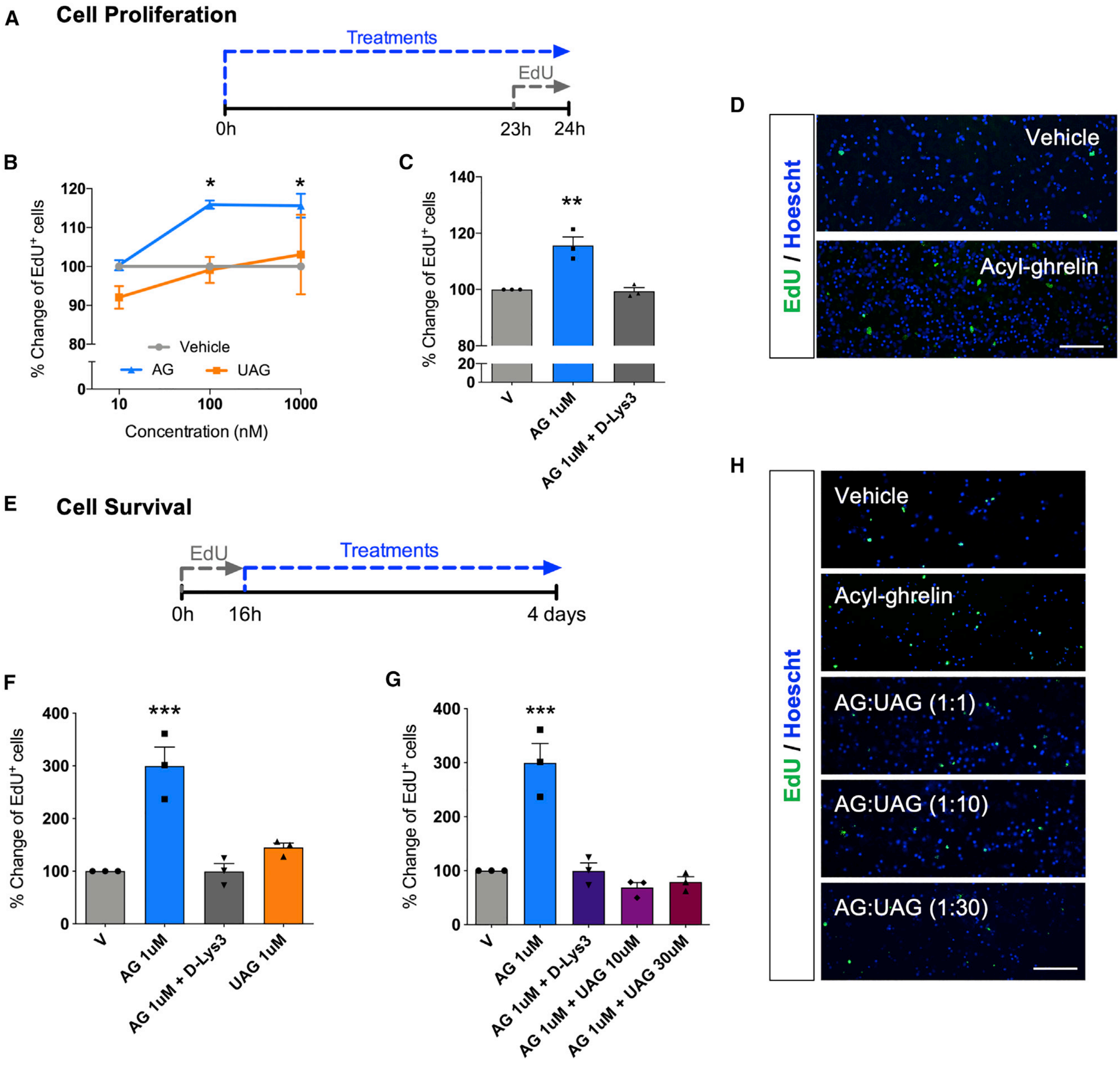

Figure 5. UAG Inhibits Acyl-ghrelin-Mediated Cell Proliferation and Survival in Primary Hippocampal Cultures

(A–C) Schematic representation of the cell proliferation assay (A). Acyl-ghrelin (AG) but not unacylated ghrelin (UAG), directly stimulated cell proliferation in a GHSR-dependent manner (B and C).

(D) Representative images of newly divided EdU^+ cells in primary hippocampal cultures.

(E) Schematic representation of the cell survival assay.

(F) AG, but not UAG, increased new cell survival in a GHSR-dependent manner.

(G) UAG inhibited the AG-mediated increase in new cell survival.

(H) Representative images of surviving EdU^+ cells in primary hippocampal cultures.

Statistical analysis performed using two-way ANOVA followed by Dunnett's post hoc test (B), one-way ANOVA followed by Dunnett's post hoc test (C, F, and G). Scale bar, 400 μm. *$p < 0.05$, **$p < 0.01$, ***$p < 0.001$ versus vehicle. Each independent experiment was performed three times, with each treatment condition performed in triplicate. Data shown are mean ± SEM.

(Figures 5A, 5B, and 5D). The proliferative effect of acyl-ghrelin was lost when the cells were cultured in the presence of the GHS-R-antagonist, [D-Lys3]-GHRP-6 (Figure 5C). In addition, we used a modified protocol to quantify cell survival, whereby dividing cells were pulsed with EdU for the first 16 h of culture, prior to acyl-ghrelin or UAG treatment (Figure 5E). Acyl-ghrelin significantly increased the survival of newborn EdU^+ cells after 4 days of treatment ($p = 0.0003$), while UAG had no effect

(p = 0.3202). Similarly, the pro-survival effect of acyl-ghrelin was lost when the cells were cultured in the presence of [D-Lys3]-GHRP-6 ($p > 0.999$) (Figure 5F). These data demonstrate that acyl-ghrelin promotes cell proliferation and survival via a direct hippocampal mechanism that is mediated by the ghrelin receptor, GHS-R. In contrast, UAG did not directly affect cell proliferation or survival, suggesting that its inhibitory effect on neurogenesis may be induced indirectly or by opposing acyl-ghrelin induced GHS-R signaling. To test whether UAG can modulate acyl-ghrelin's effect, we co-treated hippocampal cell cultures with acyl-ghrelin and UAG at equimolar (1:1) and non-equimolar doses (1:10 and 1:30), respectively. These non-equimolar doses were used to reflect the elevated level of UAG in GOAT$^{-/-}$ mice and in our UAG-treated WT mice. Notably, we report that UAG completely attenuated the pro-survival effect of acyl-ghrelin at each of the ratios tested (Figures 5G and 5H). These findings do not rule out the presence of an alternate UAG-specific receptor or steric effects that may inhibit GHS-R signaling. However, the direct antagonistic effect of UAG on acyl-ghrelin induced hippocampal cell survival, which is mediated by GHS-R, provides compelling evidence for the ability of UAG to block acyl-ghrelin-mediated GHS-R signaling in the hippocampus.

Acyl-ghrelin Increases Survival of Newborn Hippocampal Cells via BDNF in a Non-cell-Autonomous Manner

To determine whether acyl-ghrelin induces the survival of newborn cells via the action of soluble neurotrophic factors, we treated primary hippocampal cells with acyl-ghrelin and quantified the gene expression of BDNF, a known pro-neurogenic factor.[45] BDNF mRNA was significantly increased in primary hippocampal cells following treatment with acyl-ghrelin (Figure 6A). To determine whether this effect was relevant to the *in vivo* hippocampus, we treated adult mice with a single intra-peritoneal injection of acyl-ghrelin. Twenty-four hours later, we collected brain tissue for RNAScope *in situ* hybridization (ISH) analysis to show that acyl-ghrelin treatment significantly increased BDNF IXa mRNA, specifically in the rostral granule cell layer (GCL) of the DG, relative to the vehicle-treated mice ($p < 0.05$, Figures 6B and 6C). These studies confirm that acyl-ghrelin increases the expression of hippocampal BDNF *in vitro* and *in vivo*. Subsequently, we collected the conditioned media from treated primary hippocampal cultures and incubated them with a separate culture of hippocampal NSPCs[46] that do not express GHS-R (Figure 6D; Figure S7). Conditioned media collected from acyl-ghrelin treated primary hippocampal cells significantly increased the number of surviving newborn cells when incubated with hippocampal NSPCs. Notably, this pro-survival effect was completely blocked by the addition of a BDNF-neutralizing antibody to the hippocampal NSPC cultures (Figures 6E and 6F), suggesting that acyl-ghrelin supports the survival of newborn hippocampal via BDNF signaling.

The Circulating Ratio of AG:UAG Is Reduced in PDD

Mechanistically, we reasoned that as circulating levels of acyl-ghrelin and UAG have opposing actions on neurogenesis and cognition in mice and that UAG inhibits acyl-ghrelin-mediated cell proliferation and survival *in vitro*, this may be reflected in the plasma ratio of acyl-ghrelin to UAG (AG:UAG) in humans diagnosed with dementia. We therefore hypothesized that circulating AG:UAG ratios may be particularly affected in individuals diagnosed with PDD compared to a cognitively unimpaired PD group. To test this hypothesis, we recruited individuals with PD, PDD, and age-matched healthy controls to determine fasting and post-prandial levels of both acyl-ghrelin and UAG. In keeping with our pre-clinical data, we found that the plasma ratio of AG:UAG was significantly reduced in the PDD group compared to the cognitively intact PD (p = 0.0033) and control cohorts (p = 0.0145) (Figures 7A and 7B). Consistent with this finding, cognitive impairment was correlated with a reduction in plasma AG:UAG (Spearman $r^2 = 0.1164$, p = 0.0145) (Figure 7C), suggesting this ratio may be valuable as a diagnostic biomarker for human dementia. Interestingly, the cognitively intact PD group did not have reduced acyl-ghrelin levels in either the fasted state or 180 min after eating (Table S1), further supporting a specific role for ghrelin in regulating mnemonic function. Analysis of several other feeding-related (PYY, leptin), metabolism-related (insulin, GLP-1), and growth-related (GH, IGF-1) hormones, as well as cytokines (IL-6, TNF-alpha), revealed no significant differences between the groups (Figure S8).

Levels of Circulating Medium-Chain Fatty Acid Substrates for GOAT Were Unchanged in PD and PDD

To determine whether there were changes in levels of circulating GOAT substrate, we quantified medium-chain fatty acids (MCFAs) C6, C8, and C10 in fasted plasma samples from healthy control, PD, and PDD subjects. Levels of theses MCFAs, relative to the standard, nonanoic acid (C9), were unaltered in each of the three groups (Figures 7D–7F).

Ghrelin Receptor Is Expressed in the Human DG and Is Unchanged in PD and PDD

In situ BaseScope analysis of human post-mortem hippocampal brain tissue identified GHS-R1a mRNA expression within the adult human DG (Figure 7G). Notably, quantification of GHS-R1a in the hippocampal GCL from healthy control, PD, and PDD subjects revealed no significant changes in receptor expression in these groups (Figure 7H).

GOAT Expression Is Reduced in the Hippocampal GCL of PDD Brain

To determine whether the reduced AG:UAG ratio may be compensated by GOAT-mediated acylation of UAG at the hippocampus, we first performed western blot analysis of GOAT expression in hippocampal lysates from control, PD, and PDD brain. This assay demonstrated a significant decrease in GOAT immunoblot density in both PD ($p < 0.05$) and PDD ($p < 0.05$) tissue, relative to control (Figures 7I and 7J). These data are consistent with impaired acyl-ghrelin signaling across all hippocampal regions in both PD and PDD brain. For a specific assessment of GOAT expression within the GCL, we quantified the number of GOAT^{+} cells in this DG sub-region using immunohistochemistry on hippocampal sections from control, PD, and PDD brain (Figure 7K). Our data reveal a significant reduction in the number of

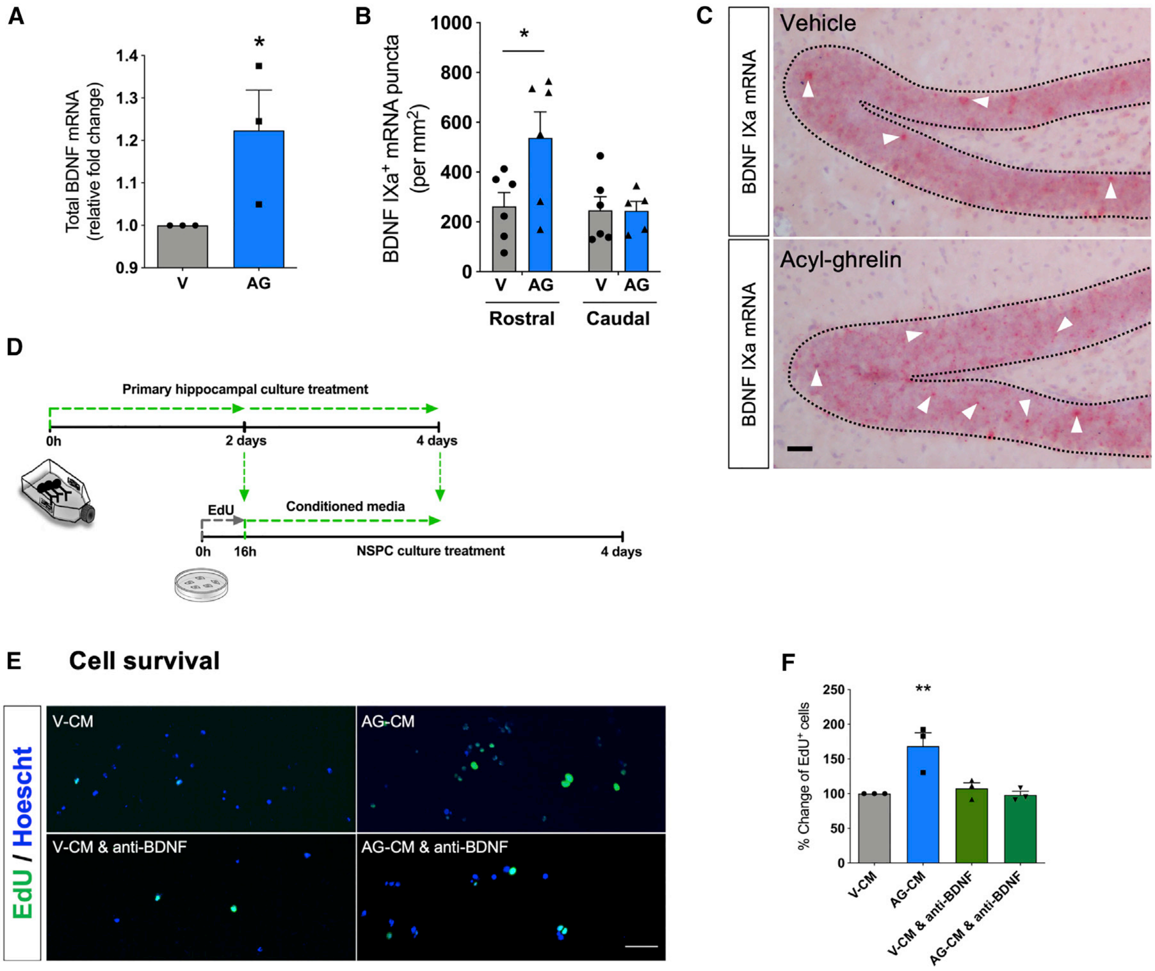

Figure 6. Acyl-Ghrelin Increases Survival of Newborn Hippocampal Cells via BDNF

(A–C) 24 h acyl-ghrelin (1 μM) increases the expression of BDNF mRNA in primary hippocampal cultures (A; $^{*}p < 0.05$, Student's t test, n = 3 mice/group) and in the GCL of the adult mouse hippocampus (B; $^{*}p < 0.05$, two-way ANOVA followed by Holm-Sidak post hoc test, n = 6 mice/group) (C; RNAScope BDNF IXa probes denoted by white arrow heads. Scale bar, 100 μm).

(D) Schematic of *in vitro* experiment to determine the non-cell-autonomous effects of acyl-ghrelin on neurogenesis. Initial treatment of primary hippocampal cultures with acyl-ghrelin, followed by transfer of conditioned media to hippocampal NSPCs and subsequent analysis of cell survival.

(E and F) Conditioned media from acyl-ghrelin (AG-CM)-treated primary hippocampal cultures increases survival of hippocampal NSPCs in a BDNF-dependent manner (E). These data suggest that acyl-ghrelin stimulates the release of BDNF from primary hippocampal cells to promote the survival of newborn hippocampal NSPCs in a non-cell-autonomous manner. Statistical analysis performed by one-way ANOVA followed by Dunnett's post hoc test. $^{**}p < 0.01$. Scale bar, 400 μm. Each independent *in vitro* experiment was performed three times, with each treatment condition performed in triplicate. Data shown are mean ± SEM.

GOAT^{+} granule layer cells specifically in PDD tissue (Figure 7L) ($p < 0.05$).

DISCUSSION

Previous findings support a role for acyl-ghrelin in improving hippocampal neurogenesis, spine remodeling, LTP, and memory function. The current study, to the best of our knowledge, demonstrates a previously unknown function for UAG in reducing hippocampal plasticity and spatial memory performance, and opposing the cognitive enhancing effects of acyl-ghrelin. Despite several studies reporting the beneficial effects of acyl-ghrelin on neurogenesis and learning, little was known about the role of UAG, the most prevalent form of ghrelin, in this context. Our results therefore identify a critical role for the post-translational modification of ghrelin in regulating neurogenesis and learning and expand our understanding of ghrelin biology in the adult hippocampus.

We report a reduction in dividing Ki67^{+} hippocampal cells and Dcx^{+} immature neurons following the genetic ablation of GOAT. These data are consistent with similar reductions in markers of neurogenesis in WT mice treated with UAG. The impairment in neurogenesis was further substantiated by a BrdU pulse-chase study, which revealed a reduction in the number of new

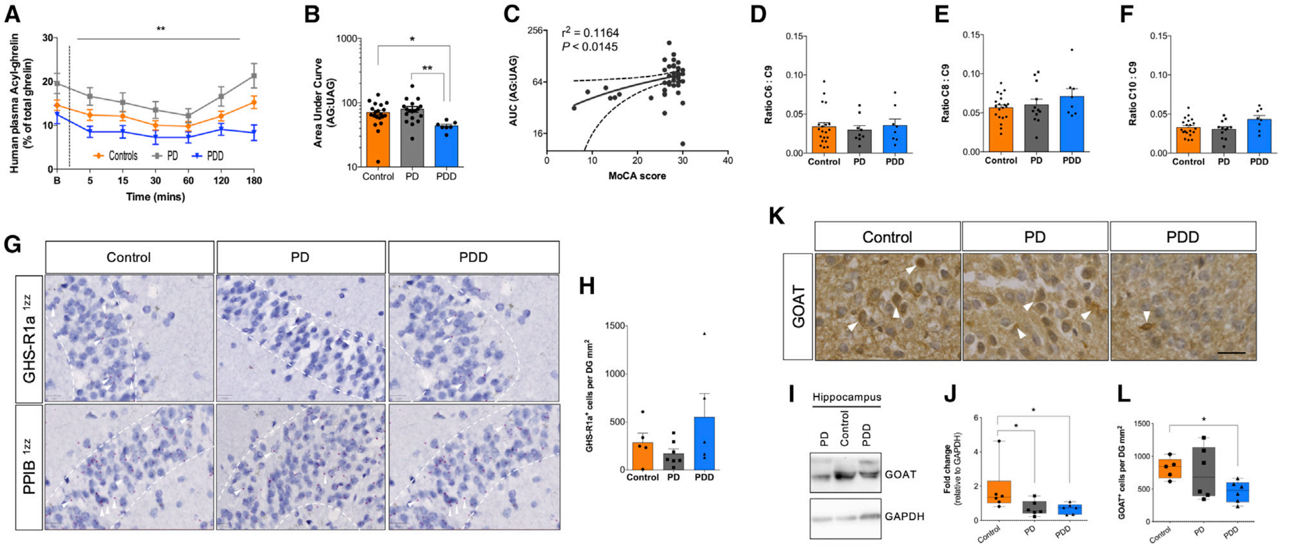

Figure 7. The Plasma Ratio of AG:UAG and Hippocampal GOAT Is Reduced in Humans with PD Dementia

(A) Plasma AG:UAG ratio in healthy controls (n = 20), PD (n = 20), and PDD (n = 8) patients under fasting and post-prandial conditions. Dotted line indicates breakfast consumption at time 0.

(B) Area-under-the-curve (AUC) values demonstrate a significant reduction in AG:UAG ratio in control versus PDD and PD versus PDD groups.

(C) Correlation of cognition (MoCA score) with plasma AG:UAG (AUC). Statistical analysis was performed by Kruskal-Wallis test with Dunn's multiple comparison and Spearman correlation analysis (two-tailed).

(D–F) Analysis of plasma free fatty acid substrates; caproic acid (C6) (D), octanoic acid (C8) (E), and capric acid (C10) (F) revealed no differences between groups. The ratio of each analyte was normalized to the internal standard, nonanoic acid (C9).

(G) BaseScope analysis of human hippocampal tissue identified ghrelin receptor (GHS-R1a^{1zz}) mRNA within the dentate gyrus (red dots, top panel, white arrowheads). Scale bar, 20 μm. Internal control mRNA probe PPIB^{1zz} is also shown (red dots, bottom panel, white arrowheads).

(H) GHS-R1a mRNA was not altered in PD or PDD brain. Statistical analysis was performed using a one-way ANOVA with the Tukey multiple-comparison test (n = 5–7/group).

(I and J) WB analysis of GOAT in hippocampal homogenates (I) identified a significant reduction in PD and PDD subjects (J) (n = 6/group).

(K and L) IHC analysis of GOAT immunoreactivity (brown, white arrowheads) in hippocampal GCL (K) identified a significant reduction GOAT$^+$ cell number in PDD subjects (L) (n = 5–6/group). Scale bar, 25 μM. Statistical analysis was performed by Kruskal-Wallis test with Dunn's multiple comparison.

All data shown are mean ± SEM. *p < 0.05, **p < 0.01.

BrdU$^+$/Dcx$^+$ adult-born immature neurons in GOAT$^{-/-}$ mice. Intriguingly, there was a reduction of new BrdU$^+$/Dcx$^-$ adult-born cells in UAG-treated WT mice and GOAT$^{-/-}$ mice, suggesting that UAG may play an important role in regulating new astroglial or new NSPCs in the hippocampus. Notably, the number of type II NSPCs was similar in both WT and GOAT$^{-/-}$ mice, suggesting that the genetic ablation of GOAT during development does not impair the generation of NSPCs in the hippocampal niche.

Analysis of other plasticity related markers also revealed changes consistent with impaired hippocampal function. These changes included a reduction in the number of c-Fos$^+$ cells within the DG in UAG-treated WT and GOAT$^{-/-}$ mice. Moreover, analysis of dendritic spines, which are essential for synaptic plasticity, revealed a reduction in the number of "stubby" spines present on hippocampal neurons from GOAT$^{-/-}$ mice. As neurogenesis, IEGs, and dendritic spines are important for brain plasticity and new memory formation, our data suggested impairments to hippocampal-dependent learning and memory. Indeed, hippocampal-dependent spatial memory testing revealed a performance deficit in GOAT$^{-/-}$ mice relative to WT mice. Recent studies suggest that acyl-ghrelin induces expression of plasticity related proteins, the NR2B subunit of the NMDA receptor,[47] and the GluA1 AMPA receptor subunit[48] to promote synaptic transmission and LTP. Our data suggest that acute 1 h acyl-ghrelin treatment is not sufficient to trigger changes that promote learning and memory. However, 7 days of daily acyl-ghrelin treatment significantly improved performance levels of GOAT$^{-/-}$ mice to WT levels. In addition, the rescue of spatial memory performance remained partially intact when tested 21 days after the final acyl-ghrelin injection. The long-term rescue of spatial memory, long after acyl-ghrelin has cleared the circulation, is consistent with longer-term changes in hippocampal plasticity such as AHN[20] and increased c-Fos-mediated transcriptional programs that support memory.[14] However, further cellular analysis of new adult-born neurons coupled with testing behavioral "pattern separation" performance, that places emphasis on distinguishing similar but distinct spatial contexts, is required to fully elucidate the behavioral consequences of the acyl-ghrelin-mediated rescue of AHN in GOAT$^{-/-}$ mice.

We recently showed in mice that the acyl-ghrelin receptor, GHS-R, is expressed on mature DG neurons but not NSPCs.[14] This finding was further substantiated by single-cell RNA

CellPress
OPEN ACCESS

sequencing (RNA-seq) of distinct hippocampal cell populations that report GHS-R expression in mature granule neurons but not in NSPCs.[49] We therefore propose that acyl-ghrelin promotes hippocampal neurogenesis in a non-cell-autonomous manner.[22,50] To validate this, we show that acyl-ghrelin treatment of hippocampal NSPCs *in vitro* had no effect on cell proliferation—a result that was consistent with the absence of detectable GHS-R expression in these cells (Figure S7). To determine whether acyl-ghrelin and UAG directly affect the hippocampus, we treated primary hippocampal cultures, which contain a mixed population of cells, including neurons and NSPCs (Figure S6). These studies demonstrated that acyl-ghrelin, but not UAG, stimulated cell division and enhanced the survival of newly divided cells in a GHS-R-dependent manner. More strikingly, UAG was able to inhibit the neurogenic effect of acyl-ghrelin *in vitro*, which is consistent with the inhibitory effect of UAG on neurogenesis in WT mice. However, while UAG inhibited the neurogenic effect of acyl-ghrelin *in vitro*, when given alone it did not reduce basal hippocampal cell division, suggesting that UAG may induce additional extra-hippocampal changes to reduce neurogenesis in mice. Nonetheless, our *in vitro* studies confirm that UAG inhibits the pro-neurogenic effect of acyl-ghrelin signaling in hippocampal cells and suggest that ghrelin, via post-translational regulation, can fine-tune neurogenesis and cognition in both positive and negative directions.

As several soluble neurotrophic factors are known to promote neurogenesis within the hippocampal niche, we reasoned that acyl-ghrelin may stimulate the production and/or release of such factors to promote neurogenesis. One such diffusible factor that promotes neurogenesis[51] and memory[45] is BDNF. Indeed, we show that BDNF mRNA was significantly increased in both primary hippocampal cells (Figure 6A) and in granule cells of the rostral, but not caudal, DG of adult mice (Figures 6B and 6C) following treatment with acyl-ghrelin. Notably, the rostral pole of the DG is linked with regulation of spatial learning and memory.[52] To determine whether acyl-ghrelin regulates the survival of newborn hippocampal cells via BDNF, we collected the conditioned media from treated primary hippocampal cultures and incubated them with hippocampal NSPCs. This culture method is well suited for testing this hypothesis as GHS-R is not expressed in NSPCs. We demonstrate that conditioned media collected from acyl-ghrelin treated primary hippocampal cells significantly increased the number of surviving newborn cells when incubated with hippocampal NSPCs. Notably, the pro-survival effect of conditioned media collected from acyl-ghrelin treated primary cells was completely blocked by the addition of a BDNF-neutralizing antibody to the hippocampal NSPC cultures (Figures 6E and 6F). These data suggest that acyl-ghrelin supports neurogenesis, at least in part, via increased hippocampal BDNF signaling.

With the findings of our pre-clinical studies in mind, the reduction in NSPC and immature neuron number in the DG of humans diagnosed with PDD[11] and the impaired performance of PD patients in an Object Pattern Separation task, we reasoned that ghrelin signaling may be impaired in PDD.[53] Therefore, we quantified plasma acyl-ghrelin in cognitively intact PD, PDD, and healthy control subjects and reported no difference between groups under fasted and fed conditions. Similarly, several circulating factors that have been reported to modulate neurogenesis and cognition, including leptin, GH, and IGF-1, were unchanged. However, quantification of total ghrelin revealed a significant reduction in the AG:UAG ratio in the PDD group compared to both PD and control groups. This is in contrast to previous reports of a reduction in circulating acyl-ghrelin in PD subjects.[54,55] This difference may be explained by our stratification of PD groups by cognitive performance. These findings are consistent with our data from pre-clinical studies demonstrating that elevated levels of UAG had detrimental effects on hippocampal neurogenesis and spatial memory.

Longitudinal studies are required to elucidate whether the AG:UAG ratio may represent a prognostic PDD biomarker. In addition, more extensive analysis of larger cohorts are needed to understand the role of AG:UAG in clinically distinct populations of patients diagnosed with dementia. As biomarkers are important for precision-medicine-based targeted therapies in dementia, the need for blood-based biomarkers to complement the high cost and invasive CSF and PET markers of amyloid-β and Tau proteins are eagerly anticipated.[56] Our findings identify a possible blood-based biomarker associated with dementia and emphasize the importance of assessing post-translational modifications in patient cohorts.

Interestingly, acyl-ghrelin treatment of GOAT$^{-/-}$ mice, which have high levels of UAG, restored learning and memory performance to control levels. We therefore speculate that raising the circulating AG:UAG ratio may ameliorate cognitive decline in PDD patients via restoration of hippocampal plasticity and pro-neurogenic signaling. Supporting this view, we show that GHS-R1a mRNA is expressed in the DG of adult humans (Figure 7G). There were no statistically significant changes across control, PD, and PDD cohorts; however, there was an almost doubling of the mean GHS-R1a mRNA expression levels in PDD versus control brain (Figure 7H). This change may reflect a homeostatic response to the reduction in plasma AG:UAG and therefore attenuated hippocampal ghrelin-signaling in PDD. These data demonstrate that the hippocampal ghrelin-receptor is present in PDD brain and that elevation of the plasma AG:UAG ratio should, in principle, result in activation of pro-neurogenic hippocampal GHS-R1a signaling. Impairments in hippocampal GHS-R1a signaling were recently identified in 5xFAD mice and in post-mortem human Alzheimer disease tissue. Notably, co-activation of GHS-R1a/DRD1 rescued synaptic and memory deficits in 5xFAD mice.[57] We show that the levels of octanoic acid, required for the post-translational formation of acyl-ghrelin, were unaltered in PDD plasma (Figures 7D–7F). Therefore, we speculate that enzyme activity for the acylation and/or de-acylation of ghrelin may be impaired in PDD leading to a reduction in the AG:UAG ratio. Of interest, recent studies indicate that ghrelin undergoes tissue-dependent acylation, including within the hippocampus, to support acyl-ghrelin signaling.[36,58] To understand the potential for UAG to undergo tissue-dependent acylation, we quantified GOAT levels in the human hippocampus. Using crude hippocampal lysates, we show a significant reduction in GOAT protein expression in both PD and PDD brain (Figures 7I and 7J). Subsequently, we quantified the number of GOAT^{+} cells within the GCL on brain tissue sections to reveal a reduction that was specific to the PDD

hippocampus (Figures 7K and 7L). Therefore, our studies suggest that a reduction in plasma AG:UAG, coupled with the inability of hippocampal cells to acylate UAG (via GOAT), leads to reduced GHS-R1a signaling in the GCL and cognitive deficits manifest in PDD. Of note, rodent toxin-based and genetic-based models of PD[16-18] have impaired hippocampal plasticity, including neurogenesis. In future, these pre-clinical models may be valuable tools in determining whether GHS-R1a agonists or compounds that inhibit ghrelin de-acylation can rescue hippocampal plasticity and cognitive function.

In summary, we describe how a post-translational modification to a circulating factor modulates, in either direction, neurogenesis and cognition in mice. In addition, the reduction in circulating AG:UAG ratio correlated with dementia in human neurodegenerative disease. The findings extend our understanding of how adult brain plasticity is regulated by circulating factors and suggest that manipulating the post-translational *acylation* of plasma ghrelin may offer therapeutic opportunities to ameliorate cognitive decline in human neurodegenerative disease.

Limitations of Study

The plasma diagnostic biomarker data presented are from relatively small cohorts. Further studies are needed, with increased sample size, to validate our findings. Also, it is not known whether the reduction in AG:UAG is specific to PDD or whether it represents a broader measure of dementia in humans. Significant additional data are required to test these possibilities in distinct dementia phenotypes. Similarly, it remains to be seen whether the use of highly sensitive analytical chemistry techniques (i.e., liquid chromatography-mass spectrometry [LC-MS]) will provide greater insight into the biochemical composition of ghrelin species in plasma from individuals diagnosed with dementia. Nonetheless, we provide a possible mechanistic link between circulating ghrelin, hippocampal function, and PDD in humans.

STAR★METHODS

Detailed methods are provided in the online version of this paper and include the following:

- KEY RESOURCES TABLE
- RESOURCE AVAILABILITY
  - Lead Contact
  - Materials Availability
  - Data and Code Availability
- EXPERIMENTAL MODEL AND SUBJECT DETAILS
  - Study Approvals
  - Animals
  - Rat Primary Hippocampal Cell Culture
  - Rat Hippocampal Stem Cells (HCN cells).
  - Human Brain
- METHOD DETAILS
  - UAG Infusion
  - Tissue Collection
  - Immunohistochemistry
  - Quantification of Labeled Cells
  - RNAscope ISH on Mouse Brain Tissue
  - Golgi-Cox Analysis of Dendritic Spines
  - Milliplex Plasma Analysis
  - Behavioral Testing
  - Proliferation and Survival Assays
  - Human Plasma Collection
  - BaseScope ISH on Human Brain Tissue
  - Immunohistochemistry of Human Brain Tissue
  - Western Blot Assay of Human Brain Tissue
  - Free Fatty Acid Analysis using Mass Spectrometry
- QUANTIFICATION AND STATISTICAL ANALYSIS

SUPPLEMENTAL INFORMATION

Supplemental Information can be found online at https://doi.org/10.1016/j.xcrm.2020.100120.

ACKNOWLEDGMENTS

The authors would like to thank Dr. Jesus A. Gutierrez (Translational Science & Technologies, Eli Lilly & Co., Indianapolis, IN, USA) for the supply of GOAT$^{-/-}$ mice and Dr. Djordje Gveric (PUK Brain Bank at Imperial College London) for the supply of post-mortem human brain tissue. We would also like to thank Mrs. Sally James (Swansea University) for assistance with confocal microscopy. The graphical abstract was created with BioRender.com. This work was funded by grants from the Medical Research Council, UK (Grant no. G0902250 to J.S.D.), St. David's Medical Foundation, UK (to J.S.D.), British Society for Neuroendocrinology, UK (to Z.B.A. and J.S.D.), NHMRC Research Fellowship, Australia (Grant no. APP1154974, to Z.B.A.), The Royal Society, UK (to J.S.D.), and the National Institute for Health Research Newcastle Biomedical Research Unit based at Newcastle Hospitals Foundation Trust and Newcastle University, UK (to D.J.B. and J.S.D.).The study involving human brain was funded by a Parkinson's UK Innovation Award, UK (to J.S.D.).

AUTHOR CONTRIBUTIONS

A.K.E.H., L.B., M.C.C., V.V.S., F.J., L.D.R., M.S., M.M., R.S., A.R., S.H.L., M.S., O.H., A.H.M., T.W., Z.B.A., and J.S.D. performed the experiments and generated the data. T.W., Z.B.A., D.J.B., and J.S.D. designed the study. J.S.D. prepared the figures and wrote the manuscript. All authors read and approved the final manuscript.

DECLARATION OF INTERESTS

The authors declare no competing interests.

Received: November 5, 2019
Revised: May 20, 2020
Accepted: September 16, 2020
Published: October 20, 2020

REFERENCES

1. Katsimpardi, L., Litterman, N.K., Schein, P.A., Miller, C.M., Loffredo, F.S., Wojtkiewicz, G.R., Chen, J.W., Lee, R.T., Wagers, A.J., and Rubin, L.L. (2014). Vascular and neurogenic rejuvenation of the aging mouse brain by young systemic factors. Science *344*, 630–634.
2. Villeda, S.A., Plambeck, K.E., Middeldorp, J., Castellano, J.M., Mosher, K.I., Luo, J., Smith, L.K., Bieri, G., Lin, K., Berdnik, D., et al. (2014). Young blood reverses age-related impairments in cognitive function and synaptic plasticity in mice. Nat. Med. *20*, 659–663.
3. Conboy, I.M., Conboy, M.J., Wagers, A.J., Girma, E.R., Weissman, I.L., and Rando, T.A. (2005). Rejuvenation of aged progenitor cells by exposure to a young systemic environment. Nature *433*, 760–764.

CellPress
OPEN ACCESS

4. Castellano, J.M., Mosher, K.I., Abbey, R.J., McBride, A.A., James, M.L., Berdnik, D., Shen, J.C., Zou, B., Xie, X.S., Tingle, M., et al. (2017). Human umbilical cord plasma proteins revitalize hippocampal function in aged mice. Nature *544*, 488–492.

5. Villeda, S.A., Luo, J., Mosher, K.I., Zou, B., Britschgi, M., Bieri, G., Stan, T.M., Fainberg, N., Ding, Z., Eggel, A., et al. (2011). The ageing systemic milieu negatively regulates neurogenesis and cognitive function. Nature *477*, 90–94.

6. Smith, L.K., He, Y., Park, J.-S., Bieri, G., Snethlage, C.E., Lin, K., Gontier, G., Wabl, R., Plambeck, K.E., Udeochu, J., et al. (2015). β2-microglobulin is a systemic pro-aging factor that impairs cognitive function and neurogenesis. Nat. Med. *21*, 932–937.

7. Rebo, J., Mehdipour, M., Gathwala, R., Causey, K., Liu, Y., Conboy, M.J., and Conboy, I.M. (2016). A single heterochronic blood exchange reveals rapid inhibition of multiple tissues by old blood. Nat. Commun. *7*, 13363.

8. Clelland, C.D., Choi, M., Romberg, C., Clemenson, G.D., Jr., Fragniere, A., Tyers, P., Jessberger, S., Saksida, L.M., Barker, R.A., Gage, F.H., and Bussey, T.J. (2009). A functional role for adult hippocampal neurogenesis in spatial pattern separation. Science *325*, 210–213.

9. Sahay, A., Scobie, K.N., Hill, A.S., O'Carroll, C.M., Kheirbek, M.A., Burghardt, N.S., Fenton, A.A., Dranovsky, A., and Hen, R. (2011). Increasing adult hippocampal neurogenesis is sufficient to improve pattern separation. Nature *472*, 466–470.

10. Aimone, J.B., Deng, W., and Gage, F.H. (2011). Resolving new memories: a critical look at the dentate gyrus, adult neurogenesis, and pattern separation. Neuron *70*, 589–596.

11. Höglinger, G.U., Rizk, P., Muriel, M.P., Duyckaerts, C., Oertel, W.H., Caille, I., and Hirsch, E.C. (2004). Dopamine depletion impairs precursor cell proliferation in Parkinson disease. Nat. Neurosci. *7*, 726–735.

12. Ally, B.A., Hussey, E.P., Ko, P.C., and Molitor, R.J. (2013). Pattern separation and pattern completion in Alzheimer's disease: evidence of rapid forgetting in amnestic mild cognitive impairment. Hippocampus *23*, 1246–1258.

13. van Praag, H., Kempermann, G., and Gage, F.H. (1999). Running increases cell proliferation and neurogenesis in the adult mouse dentate gyrus. Nat. Neurosci. *2*, 266–270.

14. Hornsby, A.K.E., Redhead, Y.T., Rees, D.J., Ratcliff, M.S.G., Reichenbach, A., Wells, T., Francis, L., Amstalden, K., Andrews, Z.B., and Davies, J.S. (2016). Short-term calorie restriction enhances adult hippocampal neurogenesis and remote fear memory in a Ghsr-dependent manner. Psychoneuroendocrinology *63*, 198–207.

15. Choi, S.H., Bylykbashi, E., Chatila, Z.K., Lee, S.W., Pulli, B., Clemenson, G.D., Kim, E., Rompala, A., Oram, M.K., Asselin, C., et al. (2018). Combined adult neurogenesis and BDNF mimic exercise effects on cognition in an Alzheimer's mouse model. Science *361*, eaan8821.

16. Winner, B., Desplats, P., Hagl, C., Klucken, J., Aigner, R., Ploetz, S., Laemke, J., Karl, A., Aigner, L., Masliah, E., et al. (2009). Dopamine receptor activation promotes adult neurogenesis in an acute Parkinson model. Exp. Neurol. *219*, 543–552.

17. Winner, B., Regensburger, M., Schreglmann, S., Boyer, L., Prots, I., Rockenstein, E., Mante, M., Zhao, C., Winkler, J., Masliah, E., and Gage, F.H. (2012). Role of α-synuclein in adult neurogenesis and neuronal maturation in the dentate gyrus. J. Neurosci. *32*, 16906–16916.

18. Winner, B., Melrose, H.L., Zhao, C., Hinkle, K.M., Yue, M., Kent, C., Braithwaite, A.T., Ogholikhan, S., Aigner, R., Winkler, J., et al. (2011). Adult neurogenesis and neurite outgrowth are impaired in LRRK2 G2019S mice. Neurobiol. Dis. *41*, 706–716.

19. Diano, S., Farr, S.A., Benoit, S.C., McNay, E.C., da Silva, I., Horvath, B., Gaskin, F.S., Nonaka, N., Jaeger, L.B., Banks, W.A., et al. (2006). Ghrelin controls hippocampal spine synapse density and memory performance. Nat. Neurosci. *9*, 381–388.

20. Kent, B.A., Beynon, A.L., Hornsby, A.K.E., Bekinschtein, P., Bussey, T.J., Davies, J.S., and Saksida, L.M. (2015). The orexigenic hormone acyl-ghrelin increases adult hippocampal neurogenesis and enhances pattern separation. Psychoneuroendocrinology *51*, 431–439.

21. Walker, A.K., Rivera, P.D., Wang, Q., Chuang, J.-C., Tran, S., Osborne-Lawrence, S., Estill, S.J., Starwalt, R., Huntington, P., Morlock, L., et al. (2015). The P7C3 class of neuroprotective compounds exerts antidepressant efficacy in mice by increasing hippocampal neurogenesis. Mol. Psychiatry *20*, 500–508.

22. Buntwal, L., Sassi, M., Morgan, A.H., Andrews, Z.B., and Davies, J.S. (2019). Ghrelin-Mediated Hippocampal Neurogenesis: Implications for Health and Disease. Trends Endocrinol. Metab. *30*, 844–859.

23. Gutierrez, J.A., Solenberg, P.J., Perkins, D.R., Willency, J.A., Knierman, M.D., Jin, Z., Witcher, D.R., Luo, S., Onyia, J.E., and Hale, J.E. (2008). Ghrelin octanoylation mediated by an orphan lipid transferase. Proc. Natl. Acad. Sci. USA *105*, 6320–6325.

24. Yang, J., Brown, M.S., Liang, G., Grishin, N.V., and Goldstein, J.L. (2008). Identification of the acyltransferase that octanoylates ghrelin, an appetite-stimulating peptide hormone. Cell *132*, 387–396.

25. Kojima, M., Hosoda, H., Date, Y., Nakazato, M., Matsuo, H., and Kangawa, K. (1999). Ghrelin is a growth-hormone-releasing acylated peptide from stomach. Nature *402*, 656–660.

26. Delhanty, P.J.D., Sun, Y., Visser, J.A., van Kerkwijk, A., Huisman, M., van Ijcken, W.F.J., Swagemakers, S., Smith, R.G., Themmen, A.P., and van der Lely, A.J. (2010). Unacylated ghrelin rapidly modulates lipogenic and insulin signaling pathway gene expression in metabolically active tissues of GHSR deleted mice. PLoS ONE *5*, e11749.

27. Matsuda, K., Miura, T., Kaiya, H., Maruyama, K., Shimakura, S., Uchiyama, M., Kangawa, K., and Shioda, S. (2006). Regulation of food intake by acyl and des-acyl ghrelins in the goldfish. Peptides *27*, 2321–2325.

28. Kumar, R., Salehi, A., Rehfeld, J.F., Höglund, P., Lindström, E., and Håkanson, R. (2010). Proghrelin peptides: Desacyl ghrelin is a powerful inhibitor of acylated ghrelin, likely to impair physiological effects of acyl ghrelin but not of obestatin A study of pancreatic polypeptide secretion from mouse islets. Regul. Pept. *164*, 65–70.

29. Fernandez, G., Cabral, A., Cornejo, M.P., De Francesco, P.N., Garcia-Romero, G., Reynaldo, M., and Perello, M. (2016). Des-Acyl Ghrelin Directly Targets the Arcuate Nucleus in a Ghrelin-Receptor Independent Manner and Impairs the Orexigenic Effect of Ghrelin. J. Neuroendocrinol. *28*, 12349.

30. Stevanovic, D.M., Grefhorst, A., Themmen, A.P.N., Popovic, V., Holstege, J., Haasdijk, E., Trajkovic, V., van der Lely, A.J., and Delhanty, P.J. (2014). Unacylated ghrelin suppresses ghrelin-induced neuronal activity in the hypothalamus and brainstem of male rats [corrected]. PLoS ONE *9*, e98180.

31. Zhao, T.-J., Liang, G., Li, R.L., Xie, X., Sleeman, M.W., Murphy, A.J., Valenzuela, D.M., Yancopoulos, G.D., Goldstein, J.L., and Brown, M.S. (2010). Ghrelin O-acyltransferase (GOAT) is essential for growth hormone-mediated survival of calorie-restricted mice. Proc. Natl. Acad. Sci. USA *107*, 7467–7472.

32. Gauna, C., Delhanty, P.J.D., Hofland, L.J., Janssen, J.A., Broglio, F., Ross, R.J., Ghigo, E., and van der Lely, A.J. (2005). Ghrelin stimulates, whereas des-octanoyl ghrelin inhibits, glucose output by primary hepatocytes. J. Clin. Endocrinol. Metab. *90*, 1055–1060.

33. Asakawa, A., Inui, A., Fujimiya, M., Sakamaki, R., Shinfuku, N., Ueta, Y., Meguid, M.M., and Kasuga, M. (2005). Stomach regulates energy balance via acylated ghrelin and desacyl ghrelin. Gut *54*, 18–24.

34. Ariyasu, H., Takaya, K., Iwakura, H., Hosoda, H., Akamizu, T., Arai, Y., Kangawa, K., and Nakao, K. (2005). Transgenic mice overexpressing des-acyl ghrelin show small phenotype. Endocrinology *146*, 355–364.

35. Sun, W., Winseck, A., Vinsant, S., Park, O.H., Kim, H., and Oppenheim, R.W. (2004). Programmed cell death of adult-generated hippocampal neurons is mediated by the proapoptotic gene Bax. J. Neurosci. *24*, 11205–11213.

36. Hopkins, A.L., Nelson, T.A.S., Guschina, I.A., Parsons, L.C., Lewis, C.L., Brown, R.C., Christian, H.C., Davies, J.S., and Wells, T. (2017). Unacylated ghrelin promotes adipogenesis in rodent bone marrow via ghrelin O-acyl transferase and GHS-R_{1a} activity: evidence for target cell-induced acylation. Sci. Rep. *7*, 45541.

37. Bayliss, J.A., Lemus, M., Santos, V.V., Deo, M., Elsworth, J.D., and Andrews, Z.B. (2016). Acylated but not des-acyl ghrelin is neuroprotective in an MPTP mouse model of Parkinson's disease. J. Neurochem. *137*, 460–471.

38. Sun, Y., Ahmed, S., and Smith, R.G. (2003). Deletion of ghrelin impairs neither growth nor appetite. Mol. Cell. Biol. *23*, 7973–7981.

39. Li, E., Chung, H., Kim, Y., Kim, D.H., Ryu, J.H., Sato, T., Kojima, M., and Park, S. (2013). Ghrelin directly stimulates adult hippocampal neurogenesis: implications for learning and memory. Endocr. J. *60*, 781–789.

40. Morgan, J.I., and Curran, T. (1991). Stimulus-transcription coupling in the nervous system: involvement of the inducible proto-oncogenes fos and jun. Annu. Rev. Neurosci. *14*, 421–451.

41. Tonegawa, S., Morrissey, M.D., and Kitamura, T. (2018). The role of engram cells in the systems consolidation of memory. Nat. Rev. Neurosci. *19*, 485–498.

42. Berrout, L., and Isokawa, M. (2012). Ghrelin promotes reorganization of dendritic spines in cultured rat hippocampal slices. Neurosci. Lett. *516*, 280–284.

43. Matsuzaki, M., Honkura, N., Ellis-Davies, G.C.R., and Kasai, H. (2004). Structural basis of long-term potentiation in single dendritic spines. Nature *429*, 761–766.

44. Kasai, H., Fukuda, M., Watanabe, S., Hayashi-Takagi, A., and Noguchi, J. (2010). Structural dynamics of dendritic spines in memory and cognition. Trends Neurosci. *33*, 121–129.

45. Ma, D.K., Jang, M.-H., Guo, J.U., Kitabatake, Y., Chang, M.-L., Pow-Anpongkul, N., Flavell, R.A., Lu, B., Ming, G.L., and Song, H. (2009). Neuronal activity-induced Gadd45b promotes epigenetic DNA demethylation and adult neurogenesis. Science *323*, 1074–1077.

46. Palmer, T.D., Takahashi, J., and Gage, F.H. (1997). The adult rat hippocampus contains primordial neural stem cells. Mol. Cell. Neurosci. *8*, 389–404.

47. Ghersi, M.S., Gabach, L.A., Buteler, F., Vilcaes, A.A., Schiöth, H.B., Perez, M.F., and de Barioglio, S.R. (2015). Ghrelin increases memory consolidation through hippocampal mechanisms dependent on glutamate release and NR2B-subunits of the NMDA receptor. Psychopharmacology (Berl.) *232*, 1843–1857.

48. Ribeiro, L.F., Catarino, T., Santos, S.D., Benoist, M., van Leeuwen, J.F., and Esteban, J.A. (2014). Ghrelin triggers the synaptic incorporation of AMPA receptors in the hippocampus. Proc. Natl. Acad. Sci. USA *111*, E149–D158.

49. Zeisel, A., Hochgerner, H., Lönnerberg, P., Johnsson, A., Memic, F., van der Zwan, J., Häring, M., Braun, E., Borm, L.E., La Manno, G., et al. (2018). Molecular architecture of the mouse nervous system. Cell *174*, 999–1014.

50. Morgan, A.H., Andrews, Z.B., and Davies, J.S. (2017). Less is more: Caloric regulation of neurogenesis and adult brain function. J. Neuroendocrinol. *29*, e12512.

51. Bekinschtein, P., Kent, B.A., Oomen, C.A., Clemenson, G.D., Gage, F.H., Saksida, L.M., and Bussey, T.J. (2013). BDNF in the dentate gyrus is required for consolidation of "pattern-separated" memories. Cell Rep. *5*, 759–768.

52. Fanselow, M.S., and Dong, H.-W. (2010). Are the dorsal and ventral hippocampus functionally distinct structures? Neuron *65*, 7–19.

53. Wesnes, K.A., and Burn, D.J. (2014). Compromised Object Pattern Separation Performance in Parkinson's Disease Suggests Dentate Gyrus Neurogenesis may be Compromised in the Condition. J Alzheimer's Dis Park *04*, 5–8.

54. Unger, M.M., Möller, J.C., Mankel, K., Eggert, K.M., Bohne, K., Bodden, M., Stiasny-Kolster, K., Kann, P.H., Mayer, G., Tebbe, J.J., and Oertel, W.H. (2011). Postprandial ghrelin response is reduced in patients with Parkinson's disease and idiopathic REM sleep behaviour disorder: a peripheral biomarker for early Parkinson's disease? J. Neurol. *258*, 982–990.

55. Song, N., Wang, W., Jia, F., Du, X., Xie, A., He, Q., Shen, X., Zhang, J., Rogers, J.T., Xie, J., and Jiang, H. (2017). Assessments of plasma ghrelin levels in the early stages of parkinson's disease. Mov. Disord. *32*, 1487–1491.

56. Hampel, H., O'Bryant, S.E., Molinuevo, J.L., Zetterberg, H., Masters, C.L., Lista, S., Kiddle, S.J., Batrla, R., and Blennow, K. (2018). Blood-based biomarkers for Alzheimer disease: mapping the road to the clinic. Nat. Rev. Neurol. *14*, 639–652.

57. Tian, J., Guo, L., Sui, S., Driskill, C., Phensy, A., Wang, Q., et al. (2019). Disrupted hippocampal growth hormone secretagogue receptor 1α interaction with dopamine receptor D1 plays role in Alzheimer's disease. Sci. Transl. Med. *11*, eaav6278.

58. Murtuza, M.I., and Isokawa, M. (2018). Endogenous ghrelin-O-acyltransferase (GOAT) acylates local ghrelin in the hippocampus. J. Neurochem. *144*, 58–67.

59. Harris, K.M., Jensen, F.E., and Tsao, B. (1992). Three-dimensional structure of dendritic spines and synapses in rat hippocampus (CA1) at postnatal day 15 and adult ages: implications for the maturation of synaptic physiology and long-term potentiation. J. Neurosci. *12*, 2685–2705.

60. Hering, H., and Sheng, M. (2001). Dendritic spines: structure, dynamics and regulation. Nat. Rev. Neurosci. *2*, 880–888.

61. Sarnyai, Z., Sibille, E.L., Pavlides, C., Fenster, R.J., McEwen, B.S., and Toth, M. (2000). Impaired hippocampal-dependent learning and functional abnormalities in the hippocampus in mice lacking serotonin(1A) receptors. Proc. Natl. Acad. Sci. USA *97*, 14731–14736.

62. Conrad, C.D., Galea, L.A.M., Kuroda, Y., and McEwen, B.S. (1996). Chronic stress impairs rat spatial memory on the Y maze, and this effect is blocked by tianeptine pretreatment. Behav. Neurosci. *110*, 1321–1334.

63. Dubois, B., Burn, D., Goetz, C., Aarsland, D., Brown, R.G., Broe, G.A., Dickson, D., Duyckaerts, C., Cummings, J., Gauthier, S., et al. (2007). Diagnostic procedures for Parkinson's disease dementia: recommendations from the movement disorder society task force. Mov. Disord. *22*, 2314–2324.

64. Bankhead, P., Loughrey, M.B., Fernández, J.A., Dombrowski, Y., McArt, D.G., Dunne, P.D., McQuaid, S., Gray, R.T., Murray, L.J., Coleman, H.G., et al. (2017). QuPath: Open source software for digital pathology image analysis. Sci. Rep. *7*, 16878.

65. Zhang, R., Brennan, M.L., Shen, Z., MacPherson, J.C., Schmitt, D., Molenda, C.E., and Hazen, S.L. (2002). Myeloperoxidase functions as a major enzymatic catalyst for initiation of lipid peroxidation at sites of inflammation. J. Biol. Chem. *277*, 46116–46122.

STAR★METHODS

KEY RESOURCES TABLE

REAGENT or RESOURCE	SOURCE	IDENTIFIER
Antibodies		
Rat anti-BrdU	BioRad	MCA6143: RRID: AB_2868611
Goat anti-Dcx (C-18)	Santa Cruz Biotechnology	sc-8066; RRID: AB_2088494
Rabbit anti-Sox2	Abcam	ab97959; RRID: AB_2341193
Mouse anti-S100β	Sigma	S2532; RRID: AB_477499
Rabbit anti-Ki67	Abcam	ab16667; RRID: AB_302459
Rabbit anti-GOAT antibody	Phoenix Pharmaceuticals	G-032-12; RRID: AB_2868614
Rabbit anti-GAPDH	Sigma	G9545; RRID: AB_796208
Rabbit anti-c-Fos	Santa Cruz	SC-52; RRID: AB_2106783
Rabbit anti-BDNF	Millipore	AB1779SP; RRID: AB_90994
Goat anti-GFAP	BioRad	AHP1468; RRID: AB_2294553
Rabbit anti-Sox2	Abcam	ab97959; RRID: AB_2341193
Mouse anti-NeuN	Millipore	MAB377; RRID: AB_2298772
Donkey anti-Goat AF-568	ThermoFisher	A11057; RRID: AB_2534104
Donkey anti-Mouse AF-568	ThermoFisher	A10037; RRID: AB_2534013
Donkey anti-rat AF-488	Life Technologies	A-21208; RRID: AB_2535794
Goat anti-rabbit AF-568	Life Technologies	A-11036; RRID: AB_10563566
Goat anti-mouse AF-488	Life Technologies	A-11001; RRID: AB_2534069
Biotinylated goat anti-rabbit	Vectorlabs	BA-1000; RRID: AB_2313606
Biotinylated donkey anti-goat	ThermoFisher	PA1-28663; RRID: AB_10980902
Biological Samples		
Hippocampal brain tissue sections (for Basescope assay).<br>Healthy controls (n = 5); Age (mean ± SD) 73.2 ± 24.27; Males 40%<br>PD (n = 7); Age (mean ± SD) 78.43 ± 5.798; Males 57.14%<br>PDD (n = 5); Age (mean ± SD) 80.6 ± 1.673; Males 40%	PUK Brain Bank at Imperial College London (ethical approval: 07/MRE09/72)	N/A
Hippocampal brain tissue sections (for IHC assay).<br>Healthy controls (n = 5); Age (mean ± SD) 73.2 ± 24.27; Males 40%<br>PD (n = 6); Age (mean ± SD) 80 ± 5.292; Males 50%<br>PDD (n = 6); Age (mean ± SD) 77.33 ± 8.14; Males 50%	PUK Brain Bank at Imperial College London (ethical approval: 07/MRE09/72)	N/A
Hippocampal brain tissue (for WB assay).<br>Healthy controls (n = 6); Age (mean ± SD) 88 ± 5.865; Males 50%<br>PD (n = 6); Age (mean ± SD) 81.83 ± 6.432; Males 66.66%<br>PDD (n = 6); Age (mean ± SD) 81.83 ± 6.432; Males 66.66%	PUK Brain Bank at Imperial College London (ethical approval: 07/MRE09/72)	N/A
Human plasma samples;<br>Healthy controls (n = 20); Age (mean ± SD) 74 ± 6.28; Males 55%<br>PD (n = 20); Age (mean ± SD) 72.2 ± 5.51; Males 55%<br>PDD (n = 8); Age (mean ± SD) 74.75 ± 5.99; Males 87.5%	Clinical Aging Research Unit, Newcastle University (ethical approval: 14/NE/0002)	N/A
Chemicals, Peptides, and Recombinant Proteins		
B27	ThermoFisher	17504
GlutaMax	ThermoFisher	35050
L-glutamine	ThermoFisher	25030081
Neurobasal media	ThermoFisher	21103
Penicillin-streptomycin-fungizone	ThermoFisher	15240062
Phosphate Buffered Saline	ThermoFisher	10010031
Poly-l-ornithine	Sigma	P4957

(Continued on next page)

Continued

REAGENT or RESOURCE	SOURCE	IDENTIFIER
Laminin	AMS Bio	3400-010-02
DMEM F12	ThermoFisher	21331
Poly-d-lysine	Sigma	P6407
Accutase	Millipore	SCR005
N2	ThermoFisher	A1370701
Trypan Blue	ThermoFisher	15250061
[D-Lys3]-GHRP-6	Tocris	1922
Acyl-ghrelin	Tocris	1465
Ethylene glycol	Sigma	324558
Glycerol	Sigma	G5516
bFGF	Peprotech	100-18B
ImmPACT® DAB	Vectorlabs	SK-4105
Sample loading buffer	BioRad	1610747
ECL Select	GE Healthcare	RPN2235
Prolong-gold anti-fade solution	Life Technologies	P36930
[D-Lys^{3}]-GHRP-6	Tocris	1922
Acyl-ghrelin (rat)	Tocris	1465
Des-octanoyl-ghrelin	Tocris	2951
Ethylene glycol	Sigma	324558
Glycerol	Sigma	G5516
bFGF	Peprotech	100-18B
AEBSF	Sigma Aldrich	A8456
Ethanol > 99.5%	Sigma Aldrich	459836
Gill's hematoxylin	Vectorlabs	H-3401-500
Vectamount mounting media	Vectorlabs	H-5000
Superfrost^{+} slides	VWR, France	631-0108
Bio-Plex Sheath Fluid	BioRad	171-000055
Vacutainer® EDTA-plasma tubes	VWR, France	6450
Critical Commercial Assays		
EdU detection kit	ThermoFisher	C10350
RNA scope 2.5 Red Assay	ACD Bio	322360
RNA scope BDNF probes	ACD Bio	461591
Milliplex-MAP 6-plex mouse cytokine magnetic bead panel kit	Millipore	SPR402
Milliplex MAP Kit - Human Metabolic Hormone Magnetic Bead Panel	Millipore	HMHEMAG-34K
EdU Click-IT assay	ThermoFisher	C10337
AllPrep DNA/RNA/Protein mini kit	QIAGEN	80204
Pierce BCA Protein Assay Kit	ThermoFisher	23227
VectaStain Elite ABC-HRP Kit	Vectorlabs	PK-6100
Human Ghrelin (Total) ELISA	Millipore	EZGRT-89K
Human Ghrelin (Active) ELISA	Millipore	EZGRA-88K
Human IGF-1 DuoSet ELISA	R&D Systems	DY291
Human GH DuoSet ELISA	R&D Systems	DY1067
BaseScope Reagent Kit - RED	Advanced Cell Diagnostics	322900
BaseScope Probe – BA-Hs-GHSR-tv1a-E1E2	Advanced Cell Diagnostics	709121
FD Rapid GolgiStain Kit	FD Neurotechnologies Inc.	PK401A
Experimental Models: Cell Lines		
Rat hippocampal primary cells	ThermoFisher	A1084101
Rat neural stem/progenitor cell-line	Hsieh Lab	Palmer et al.[46]

(*Continued on next page*)

Continued

REAGENT or RESOURCE	SOURCE	IDENTIFIER
Experimental Models: Organisms/Strains		
GOAT$^{-/-}$ mice: 12-week old male mice on a C57BL/6 genetic background were maintained at Monash University, Australia, with approval from the Monash University Animal Ethics Committee.	Regeneron Pharmaceuticals (Tarrytown, NY, USA)	Bayliss et al.[37]
GOAT$^{-/-}$ mice: 6-month old male mice on a C57BL/6 genetic background were maintained at Cardiff University, UK, with approval from the UK Animals (Scientific Procedures) Act 1986.	Taconic Farms (Hudson, NY, USA)	Hopkins et al.[36]
Ghrelin$^{-/-}$ mice 5-month old female mice on a C57BL/6 genetic background were maintained at Cardiff University, UK, with approval from the UK Animals (Scientific Procedures) Act 1986.	Dr Yuxiang Sun lab (Texas A&M, TX, USA)	Sun et al.[38]
Oligonucleotides		
Total BDNF mRNA oligos (NCBI: M61178) F: CGAGAGGTCTGACGACGACG	PrimerDesign, UK	This paper
Total BDNF mRNA oligos (NCBI: M61178) R: GCGTCCTTATGGTTTTCTTCGTTG	PrimerDesign, UK	This paper
Software and Algorithms		
GraphPad Prism 8.0	https://www.graphpad.com/	N/A
ImageJ	https://www.imagej.nih.gov/	N/A
QuPath	https://qupath.github.io	N/A
Bio-Plex Manager v4.1	Bio-Rad	N/A
ImageLab Software v4.1 (ChemiDoc XRS)	BioRad	N/A
Other		
In Cell Analyzer 2000	GE	N/A
Nikon Eclipse 50i microscope	Nikon	N/A
Fluorescent microscope (Axioscope)	Zeiss	N/A
Confocal microscope (LSM710 META).	Zeiss	N/A
Axio Scan.Z1	Zeiss	N/A
LTQ Orbitrap XL	Thermo Scientific	N/A
CM1900 Cryostat	Leica	N/A
Freeezing-stage microtome (MicroM)	ThermoScientific	N/A
POLARstar Omega plate reader	BMG Labtech	N/A
Bioplex-200 System	Bio-Rad	N/A

RESOURCE AVAILABILITY

Lead Contact

Further information and requests for resources and reagents should be directed to and will be fulfilled by the Lead Contact, Jeff Davies (jeff.s.davies@swansea.ac.uk).

Materials Availability

This study did not generate unique reagents.

Data and Code Availability

This study did not generate datasets or code.

EXPERIMENTAL MODEL AND SUBJECT DETAILS

Study Approvals

The animal procedures described, including those involving genetically modified animals, conformed to the UK Animals (Scientific Procedures) Act 1986 and the Monash University Animal Ethics Committee guidelines. All procedures complied with the NC3Rs

ARRIVE Guidelines on reporting *in vivo* experiments. The study involving humans was approved by Local Ethical Review (14/NE/0002; IRAS project ID: 141456) at Newcastle University, consistent with the Mental Capacity Act 2005.

Animals

Six-month old homozygous GOAT null ($GOAT^{-/-}$) mice and their WT (C57BL/6) littermate controls[23] were imported from Taconic Farms (Hudson, NY) and housed in the JBIOS animal facility (Cardiff University) under standard laboratory conditions (12h L:D cycle) with food and water available *ad libitum.* Six-month old Ghrelin null ($Ghrelin^{-/-}$) mice and their WT littermates[29] were bred from heterozygous x heterozygous mating in the animal facility at Cardiff University under standard conditions (as above). Founder stock were a kind gift from Prof. Yuxiang Sun (Texas A&M University, College Station, USA). A second genetic model of GOAT ablation[24] was provided by Regeneron Pharmaceuticals. This $GOAT^{-/-}$ line was generated using Velocigene technology. The GOAT gene sequence (ATG-stop) was replaced with a lacZ reporter gene using the target vector, bacterial artificial chromosome (BAC). These mice originated from C57BL/6/129 targeted embryonic stem cells and mice were backcrossed onto a C57BL/6 mice background. Mice were kept in standard laboratory conditions at Monash University with free access to food (chow diet, cat no. 8720610 Barastoc stockfeeds, Victoria Australia), and water at 23°C in a 12-hour light/dark cycle and were group-housed to prevent isolation stress, unless otherwise stated. The allocation of mice into groups was performed in a randomized manner and data collection was performed by a person blind to the treatment conditions.

Rat Primary Hippocampal Cell Culture

Primary rat hippocampal cultures from the hippocampi of day-18 Fisher 344 rat embryos were grown in neurobasal media (ThermoFisher, 21103), supplemented with B27 supplement (ThermoFisher, 17504), 200mM GlutaMax (ThermoFisher, 35050) and 25μM L-glutamine (ThermoFisher, 25030081). Tissue culture plastic was coated with poly-d-lysine (Sigma, P6407) at a concentration of 4.5μg/cm^2. Cells were maintained at 37°C in a 5% CO_2 humidified incubator.

Rat Hippocampal Stem Cells (HCN cells).

These cells, herein referred to as Neural Stem/Progenitor Cells (NSPCs), initially isolated and cloned from Fisher 344 rats, were a kind gift from Prof Jenny Hsieh's lab (University of Texas, San Antonio, USA). These cells were cultured in DMEM F12 (ThermoFisher, 21331), supplemented with N2 supplement (ThermoFisher, A1370701), GlutaMax (ThermoFisher, 35050061), Penicillin-streptomycin-fungizone (ThermoFisher, 15240062) and 20ng/ml bFGF/FGF2 (Peprotech, 100-18B). Tissue culture plastic was coated with 10 μg/ml Poly-l-ornithine (PLO) (Sigma, P4957) and 5 μg/ml laminin (AMS Bio, 3400-010-02). Cells were maintained at 37°C in a 5% CO_2 humidified incubator.

Human Brain

The brain tissue was collected by the PUK Brain Bank at Imperial College London with ethical approval (07/MRE09/72) and was used in this study with consent following peer review. A total of 17 subjects were included in the BaseScope analysis. Post-mortem hippocampal tissue sections were prepared from formalin fixed frozen tissue, cryo-sectioned (6-8um thick) and mounted onto Superfrost glass slides. Brain tissue was prepared from healthy controls (n = 5), with no evidence of degenerative disease or cognitive decline, participants diagnosed with PD (n = 7) and participants diagnosed with PDD (n = 5). All cases had a post-mortem interval of < 24h. Upon arrival, sections were stored at −70°C and sections from across the anterior-posterior extent of the hippocampus were visually determined for use in subsequent BaseScope assays.

METHOD DETAILS

UAG Infusion

Each mouse[23] was fitted with an indwelling jugular vein catheter connected to a subcutaneous osmotic mini-pump (ALZET model 1007D) primed to deliver vehicle (sterile isotonic saline containing BSA (1mg/ml) and heparin (5U/ml) at 0.5μl/h) or UAG (48ug/day) under isoflurane anesthesia. One day later all mice received an injection of thymidine analog, BrdU (50mg/kg i.p), to label dividing cells. After seven days mice were re-anesthetized and killed by decapitation, the brains being excised whole and processed for immunohistochemistry (IHC) as described below.

Tissue Collection

Whole brain was removed and immediately fixed by immersion in 4% paraformaldehyde (PFA) in 0.1M phosphate buffer (pH 7.4) for 24h at 4°C unless stated otherwise. Subsequently brains were cryoprotected in 30% sucrose solution (until sunk). Coronal sections (30 μm) were cut into a 1:6 series along the entire rostro-caudal extent of the hippocampus using a freezing-stage microtome (MicroM, ThermoScientific) and collected for IHC. All IHC was performed on free-floating sections at room temperature unless stated otherwise. A sub-set of 12-week old $GHSR^{-/-}$ mice (see Fig.S3) were terminally anesthetized and intra-cardially perfused with 4% PFA prior to cryoprotection, as described above.

Immunohistochemistry

For immunofluorescent analysis of $BrdU^+/Dcx^+$, sections were washed three times in PBS for 5 minutes, permeabilized in methanol at −20°C for 2 minutes and washed (as before) prior to pre-treatment with 2N HCl for 30 minutes at 37°C followed by washing in 0.1 M borate buffer, pH8.5, for 10 minutes. Sections were washed as before and blocked with 5% normal donkey serum (NDS) in PBS plus 0.1% triton (PBS-T) for 60 minutes at room temperature. Sections were incubated overnight at 4°C in rat anti-BrdU (1:400, AbD Serotec) and goat anti-Dcx (1:200, Santa Cruz Biotechnology, USA) diluted in PBS-T. Tissue were washed as before and incubated in donkey anti-rat AF-488 (1:500, Life Technologies, USA) and donkey anti-goat AF-568 (1:500, Life Technologies, USA) in PBS-T for 30 minutes in the dark. After another wash sections were mounted onto $Superfrost^+$ slides (VWR, France) with prolong-gold anti-fade solution (Life Technologies, USA).

For immunofluorescent analysis of Sox2, sections were treated as above with the exception of antigen retrieval being performed in sodium citrate at 70°C for 1h (rather than 2N HCl or borate buffer) with subsequent blocking in 5% NGS. Immunoreactivity was detected using rabbit anti-Sox2 (1:500, ab97959, Abcam) and goat anti-rabbit AF-568 (Life Technologies, USA). Nuclei were counterstained with Hoechst prior to mounting as described above.

For immunofluorescent analysis of $Sox2^+/ S100\beta^+$, sections were washed three times in PBS for 5 minutes, permeabilised in methanol for 2 minutes at −20°C and washed again (as above). Antigen retrieval was performed with sodium citrate buffer for 1 hour at 70°C, sections were washed as before and subsequently blocked with 5% normal goat serum (NGS) in PBS+0.1% Triton-X (PBS-T) for 1 hour at room temperature. Sections were incubated overnight at 4°C in rabbit anti-Sox2 (1:1000, ab97959, Abcam) diluted in PBST. Sections were washed as before and incubated in goat anti-rabbit AF-568 (1:500, Life Technologies) in PBST for 30 minutes at room temperature, in the dark. Following another wash step, sections were incubated for 1 hour at room temperature with mouse anti-S100β (1:1000, S2532, Sigma) in PBST. Following another set of washes, sections were incubated in goat anti-mouse AF-488 (1:500, Life Technologies) in PBST for 30 minutes at room temperature and protected from light. After a final wash sections were mounted onto $Superfrost^+$ Plus slides (VWR) with Prolong-gold anti-fade mounting solution (Invitrogen) and coverslipped.

For DAB-immunohistochemical analysis of Ki67, Sox2 and DCX labeling, sections were washed in 0.1M PBS (2x10mins) and 0.1M PBS-T (1x10 mins). Subsequently, endogenous peroxidases were quenched by washing in a PBS plus 1.5% H_2O_2 solution for 20 minutes. Sections were washed again (as above) and incubated in 5% NGS (NDS for DCX) in PBS-T for 1h. Sections were incubated overnight at 4°C with rabbit anti-Ki67 (1:500, ab16667, Abcam), rabbit anti-Sox2 (1:1000, ab97959, Abcam) or goat anti-DCX (1:200 Santa Cruz Biotechnology, USA), in PBS-T and 2% NGS (NDS for DCX) solution. Another wash step followed prior to incubation with biotinylated goat anti-rabbit (1:400 Vectorlabs, USA) for Ki67 and Sox2 or biotinylated donkey anti-goat (1:400 Vectorlabs, USA) for DCX, in PBS-T for 70 minutes. The sections were washed and incubated in ABC (Vectorlabs, USA) solution for 90 minutes in the dark prior to another two washes in PBS, and incubation with 0.1M sodium acetate pH6 for 10 minutes. Immunoreactivity was developed in nickel-enhanced DAB solution followed by two washes in PBS. Sections were mounted onto superfrost+ slides (VWR, France) and allowed to dry overnight before being de-hydrated and de-lipified in increasing concentrations of ethanol. Finally, sections were incubated in Histoclear (2x3 mins; National Diagnostics, USA) and coverslipped using Entellan mounting medium (Merck, USA). Slides were allowed to dry overnight prior to imaging.

For DAB-immunohistochemical analysis of c-Fos labeling, sections were washed and endogenous peroxidases quenched as before. Sections were washed again (as above), before antigen retrieval in sodium citrate at 70°C for 1h and subsequent blocking in 5% NGS in PBS-T for 1h. Sections were incubated overnight at 4°C with rabbit anti-c-Fos (1:4000, SC-52, Santa Cruz, USA) in PBS-T and 2% NGS solution. Another wash step followed prior to incubation with biotinylated goat anti-rabbit (1:400 Vectorlabs, USA) in PBS-T for 70 minutes. The sections were washed and incubated in ABC (Vectorlabs, USA) solution for 90 minutes in the dark prior to another round of washing (as above) and subsequent tyramide signal amplification. Following incubation with biotinylated tyramine (1:100) in PBS-T plus 0.1% H_2O_2 for 10 min, sections were washed in 0.1M PBS (1x10 mins) and 0.1M PBS-T (2x10 mins), before a second 90 minutes ABC (Vectorlabs, USA) incubation, which was again performed in the dark. Sections were then washed in 0.1M PBS (2x10 mins) and incubated with 0.1M sodium acetate pH6 for 10 minutes. Immunoreactivity and tissue processing was performed as described above.

Quantification of Labeled Cells

Immuno-stained brain tissue was imaged by light microscopy (Nikon 50i) (for DAB), fluorescent microscopy (Axioscope, Zeiss) or confocal microscopy (LSM710 META, Zeiss). Immunofluorescent cells were manually counted bilaterally through the z axis using a × 40 objective and throughout the rostro-caudal extent of the granule cell layer (GCL). DAB-immunolabelled cells were quantified using ImageJ software. Resulting numbers were divided by the number of coronal sections analyzed and multiplied by the distance between each section to obtain an estimate of the number of cells per hippocampus (and divided by 2 to obtain the total per DG). For quantification of immunoreactivity in $GOAT^{-/-}$ mice[24] where there were only rostral DG sections available, cell number was divided by the DG area and expressed as counts per mm^2. All analyses were performed blind to genotype and treatment.

RNAscope ISH on Mouse Brain Tissue

RNAscope is a proprietary method of *in situ* hybridization (ISH) to visualize single RNA molecules per cell. The assay was performed according to the manufacturer's instructions, including all buffers if not otherwise stated (ACD Bio, RNAscope® 2.5 Red Assay, 322360). Briefly, male C57Bl6 mice (11-13 weeks old) were treated with acyl-ghrelin (300 μg/kg; i.p at 11.00h) before brains were

collected 24h later for RNAscope assay. Coronal sections (30 μm) were cut using a freezing-stage microtome (MicroM, ThermoScientific) along the entire rostro-caudal extent of the hippocampus and collected in a 1:12 series, immersed in cryoprotectant (0.1M PBS containing 30% ethylene glycol, 20% glycerol) and stored at −80°C until use. Sections were first washed with TBS-0.1%Tween20 (TBST) to remove residual cryoprotectant, followed by incubation in TBST-1.5% H_2O_2 for 20 minutes at RT. The sections were subsequently rinsed in PBST and mounted onto SuperFrost Plus slides. Next, the slides were dehydrated in an ethanol gradient (70%–100%) for 3 minutes each, then baked in a dry oven for 60 minutes at 60°C. Target retrieval was performed by incubation in target retrieval buffer (1X) in a steamer for 15 minutes, before washing with 100% ethanol. Pre-treatment protease digestion was performed using the protease plus at 40°C for 30 minutes in the HybEz oven rack. After this, slides were incubated with the probes: BDNF IXa (461591), positive control PPIB (313911), or the negative control DapB (310043), for 2 hours at 40°C. After this, slides were washed with wash buffer (1X) and left in SSC buffer (5X) at RT overnight. Signal amplification was performed with ACD bio amplification reagents (AMP1-6) in the HybEz oven. The stain was developed for 10 minutes using the Fast-RED A & B reagents (60:1). 50% Haematoxylin was used as a counter-stain to enhance visualization and contrast of the RNA puncta. VectaMount was added per slide prior to coverslipping. Images were acquired using a light microscope (Nikon Eclipse 50i, × 10 magnification) and analyzed using the ImageJ software to quantify puncta per mm^2 of GCL.

Golgi-Cox Analysis of Dendritic Spines

Impregnation of WT and GOAT$^{-/-}$ mouse brains with Golgi-Cox solution was performed using the FD Rapid GolgiStain™ Kit (PK401A, FD Neurotechnologies Inc.) according to the manufacturer's instructions. Briefly, freshly dissected brains were rinsed with Milli-Q water to remove residual blood. The tissue was immersed in impregnation solution (equal volumes solution A and solution B) and stored, in the dark, at room temperature for 2 weeks. Impregnation solution was replaced with fresh solution after the first 24h. The tissue was transferred into solution C and stored, in the dark, at room temperature for 1 week. Solution C was replaced with fresh solution after the first 24h of immersion. Brain tissue was subsequently frozen by slowly dipping the tissue into pre-cooled isopentane, on dry-ice, for a few seconds. The tissue was placed on dry-ice for 1 minute, before being wrapped in foil and stored at −80°C.

Golgi-Cox impregnated brains were sectioned using a Leica CM1900 Cryostat (chamber temperature set to −15°C; cutting stage temperature set to −10°C) at a thickness of 120μm and mounted onto glass slides that were rinsed in Solution C. Slides were dried overnight at room temperature and protected from light. The slides were rinsed 2x with Milli-Q water, for 4 minutes each, before being placed in a mixture of 1 part Solution D, 1 part Solution E and 2 parts Milli-Q water (DEQ solution) for 10 minutes. Slides were dehydrated in a series of 50%, 75% and 95% EtOH for 4 minutes each, followed by 4x 4 minute incubations in 100% EtOH. Subsequently, the slides were delipified in Histoclear 3x for 4 minutes, and coverslipped using Entellan mounting media.

Dendritic spines were analyzed using the Nikon Eclipse 50i microscope using a Nikon Plan 100x/1.25 oil objective. The images were subsequently processed using ImageJ software in order to manually count dendritic spines, selected from secondary branches of apical dendrites. Spines were categorised into the following groups; mushroom, thin spine, stubby spine, branched and filopodium, as described[59,60]. For each genotype, 4-8 dendritic segments per mouse were analyzed. The spine density was presented as the number of spines per mm dendritic length.

Milliplex Plasma Analysis

Mouse plasma was analyzed using the Milliplex-MAP 6-plex mouse cytokine magnetic bead panel kit (Cat #SPR402), to analyze the cytokines IL-6, eotaxin, fractakine, IL-10, RANTES and TNFα. The assay was performed according to the manufacturer's guidelines. All reagents were brought to room temperature (RT) before use in the assay. Plasma samples were thawed at 4°C and diluted 1:2 with assay buffer. Antibody-immobilized beads were sonicated for 30 s and vortexed for 1 minute. 60μl from each antibody-bead vial was added to the mixing bottle provided and the final volume made up to 3ml with assay buffer. Wash buffer (WB) was prepared by mixing 60ml 10X WB with 540ml deionized water. Serum matrix solution was prepared by adding 2ml assay buffer to the lyophilized serum matrix. Subsequently, the mouse cytokine standard cocktail (Cat #MXM8070) was reconstituted with 0.25ml deionized water and a 1:5 serial dilution was made.

WB (200μl) was added to each well of the 96-well plate before it was sealed and agitated for 10 minutes at room temperature. The WB was removed and 25μl of each standard or control was added to the appropriate wells and 25μl of assay buffer to the sample wells. Next, 25μl of serum matrix was added to the background, standards and control wells, and 25μl of diluted plasma sample was added into the sample wells. Following this, the mixing bottle containing the antibody-bead mixture was vortexed and 25μl was added to each well. The plate was sealed, wrapped in foil and incubated at 2-8°C on a plate shaker for 16-18 hours.

After incubation, the well contents were removed (using a handheld magnet) and the wells washed twice with 200μl WB. Subsequently, 25μl of detection antibodies were added to each well, the plate was then sealed, covered with foil and incubated for 1h at room temperature, with agitation. Next, 25μl Streptavidin-Phycoerythrin was added to each well and incubated for 30 minutes as before. Finally, with a hand-held magnet, well contents were removed and the plate was washed twice with 200μl WB. 150μl of sheath fluid (BioRad Bio-Plex Sheath Fluid, Cat #171-000055) was added to each well for 5 minutes with agitation. The plate was assessed using a Bio-Rad Bioplex-200 System with Bio-Plex Manager 4.1 software.

Behavioral Testing

To determine whether the neurochemical deficits observed in GOAT$^{-/-}$ mice resulted in impaired hippocampal-dependent spatial memory and whether this could be rescued by acyl-ghrelin, adult 12 week-old WT and GOAT$^{-/-}$ mice (n = 6/group) were given daily injections of either saline or acyl-ghrelin (300 μg/kg i.p) for 1 or 7 days prior to analysis of spatial memory using a Y-maze. This dose of acyl-ghrelin was chosen as it has previously been shown to increase food intake. Injections were performed daily between 9-10am when mice were in a fed state. A modified Y-maze task was used to assess spatial memory performance on day 1, 7 or 28 days after the first acyl-ghrelin injection. All tests were performed in an experimental room with sound isolation and dim light. The animals were carried to the test room for at least 1 hour of acclimation. Behavior was monitored using a video camera positioned above the apparatuses and the videos were later analyzed by an experienced blinded researcher using video tracking software (CleverSys Inc, Reston, VA, USA). The modified Y-maze measures spatial memory, as spatial orientation cues facilitate rodents to explore a novel arm rather than returning to a previously visited arm[61]. This ethologically relevant test is based on the rodents' innate curiosity to explore novel areas and was chosen in these studies specifically because it does not require negative or positive reinforcers, such as food rewards, as ghrelin is known to affect food intake and motivation. In addition, it has been validated as a hippocampal relevant spatial task[62], with impaired performance in different models of hippocampal damage. We used a Y-shaped gray Perspex maze (30 cm x 10 cm x 16 cm) and each arm could be isolated by blocking entry with a sliding door. Sawdust from a mouse's home cage lined the maze during the trials and extra maze cues on the walls were placed 30-40 cm from the end of the arms to provide spatial orientation cues. Behavior was tested across two trials, the first of which had one arm of the maze blocked off. Mice were allowed to explore the reduced maze for 10 minutes and then returned to their home cage. The second trial was conducted 30 minutes after the first trial and both arms of the maze were opened. Mice were placed in the start (home) arm and allowed to explore the full maze for 5 minutes. All behaviors were recorded and analyzed using tracking software. Novel arm exploration was recorded when all four feet of each mouse entered the novel arm. The apparatus was cleaned with 80% ethanol between each trial and each animal.

Proliferation and Survival Assays

To determine the effect of ghrelin on proliferation, primary hippocampal cells were seeded at a density of $5x10^4$ cells per well for 24h with the appropriate treatments (acyl-ghrelin or UAG). To assess whether this proliferative affect was mediated by GHS-R, cells were incubated with GHS-R antagonist D-Lys3 (1μM) 0.5h before acyl-ghrelin incubation. For the final 1h of treatments, half the media was removed and incubated with EdU (well concentration 10μM), prior to fixation with 4% PFA. To determine the effect of acyl-ghrelin on survival, primary hippocampal cells were seeded at a density of $5x10^4$ cells per well for 16h in the presence of 10 μM EdU. The next day cells were rinsed thoroughly with PBS and incubated with appropriate treatments for 4 days, before being fixed with 4% PFA. Proliferation/survival were assessed by EdU Click-IT assay (ThermoFisher C10337), counting the number of EdU-positive cells as a proportion of Hoescht stained cells. Cells were imaged using a × 20 objective (InCell Analyzer, GE Healthcare) with 9 fields of view per well. Each independent experiment was performed three times, with each treatment condition performed in triplicate.

To determine the effect of ghrelin and conditioned media on cell proliferation and survival the adult rat hippocampal stem cell line (HCN) was used. These cells, herein referred to as Neural Stem/Progenitor Cells (NSPCs), initially isolated and cloned from Fisher 344 rats were a kind gift from Prof Jenny Hsieh's lab (University of Texas, San Antonio, USA), were cultured in DMEM F12 (ThermoFisher, 21331), supplemented with N2 supplement (ThermoFisher, A1370701), GlutaMax (ThermoFisher, 35050061), Penicillin-streptomycin-fungizone (ThermoFisher, 15240062) and 20ng/ml bFGF/FGF2 (Peprotech, 100-18B). Tissue culture plastic was coated with 10 μg/ml Poly-l-ornithine (PLO) (Sigma, P4957) and 5 μg/ml laminin (AMS Bio, 3400-010-02). Cells were maintained at 37°C in a 5% CO_2 humidified incubator.

To examine the effect of conditioned media on the survival of newborn cells, NSPCs were seeded at a density of $5x10^4$ cells per well in a 96-well PLO/Laminin coated plate and incubated with EdU (10 μM) for 16h to label dividing cells. This time point coincided with the end of primary hippocampal cells being treated with vehicle or acyl-ghrelin (1 μM) for 2 days. The NSPCs were washed three times with PBS to remove residual media, and the vehicle (V-CM) or acyl-ghrelin (AG-CM) treated conditioned media was transferred from the primary hippocampal cells to the NSPC cultures and incubated for 2 days. During this time, primary hippocampal cells were treated with vehicle or acyl-ghrelin for a further 2 days. At this point the conditioned media was, once again, applied to the NSPCs and incubated for a further 2 days. Following exposure to conditioned media for a total of 4 days, the NSPCs cells were fixed with 4% PFA. The survival assay was performed in triplicate and assessed via the EdU Click-iT assay, as described above. Analysis was performed using ImageJ software, with the number of EdU^+ cells expressed as a proportion of $DAPI^+$ cells.

Human Plasma Collection

All procedures involving human participants were performed at the Clinical Aging Research Unit, Newcastle University, with appropriate ethical approvals. 48 adults aged 60-85 were recruited; healthy controls (HC) (n = 20), PD (n = 20) and PDD according to level 1 Movement Disorder Task Force criteria for the diagnosis of PDD[63] (n = 8). Montreal Cognitive Assessment (MoCA) was ≥ 26/30 for HC and PD, and ≤ 25/30 for PDD. Participants with unexplained weight loss, obesity, BMI < 18 or > 30, diabetes, gastrointestinal disease, smoking, deep brain stimulation or non-selective anticholinergic medication were excluded. Participants were tested fasted and off PD medication. Blood was drawn in the fasted state (0) and at 5, 15, 30, 60, 120 and 180 minutes following a standard breakfast.

Whole blood was collected into Vacutainer® EDTA-plasma tubes (Cat No: 6450) up to a volume of 7ml per tube and inverted gently to prevent coagulation. Whole blood was transferred immediately to 3 cold centrifuge tubes; the first tube was used for quantification of ghrelin and contained AEBSF (2mg/ml) to prevent proteinase activity. The second tube was used for analysis of insulin, leptin, PYY, IL-6, GLP-1 (active) and TNFα. These 2 separate tubes were centrifuged at 2000xg for 15 minutes at 4°C, resulting in 3 layers (top plasma layer, mid white layer of leukocytes and a bottom layer of red blood cells). The top layer of plasma was carefully transferred into sterile tubes (200μl aliquots), labeled with the date and identification number, and stored at −80°C. The third tube retained platelet-rich plasma for the analysis of IGF-1 and GH, this fraction of blood was spun at 1000xg for 15 minutes at 4°C, aliquoted and stored as stated above.

Blood samples for assessing acyl-ghrelin were treated with 4-(2-Aminoethyl)-benzenesulfonyl fluoride (A8456, Sigma Aldrich) to prevent de-acylation and analyzed using a multiplex assay (Milliplex MAP Kit - Human Metabolic Hormone Magnetic Bead Panel HMHEMAG-34K, Millipore), with additional beads to quantify leptin, insulin, IL-6, TNFα, PYY and GLP-1 active. Total ghrelin was analyzed by ELISA (EZGRT-89K Millipore). For IGF-1 and GH, platelet-rich plasma samples were analyzed using Human IGF-1 DuoSet ELISA (cat. No. DY291, R&D Systems), and Human GH DuoSet ELISA (Cat. No. DY1067, R&D Systems), using half-volume Nunclon Microwell 96-well plates. Area under the curve (AUC) was calculated for each analyte, however, plasma samples that were below the standard range for GH and IGF-1 were excluded. Outliers were identified using the ROUT test (Q = 1%) and removed prior to analysis using a Kruskall-Wallis test with Dunn's post hoc multiple comparison.

BaseScope ISH on Human Brain Tissue

Sections were thawed at RT for five minutes, fixed with 10% NBF (Sigma Aldrich) for 1 h, then washed twice with PBS before dehydration in increasing concentrations of ethanol. Sections were baked at 60°C for 30min to improve tissue adhesion to the glass surface. BaseScope assay (Advanced Cell Diagnostics) was performed according to supplier guidelines. Pre-treatment conditions were optimized as follows; Hydrogen peroxide for 10 min at RT; target retrieval reagent for 15 min at 100°C; dehydration in ethanol 100% (Sigma Aldrich) for 3min. Sections were dried completely for 20 min at 60°C, then protease IV was added to the dried slides for 30 min at 40°C. Custom BaseScope probes targeting human GHS-R1a were applied for 2 h at 40°C as follows; for every case, sections were incubated with negative (DABP) and positive (PPIB) probes as well as with GHS-R1a probes (Cat.no. 709121). After incubation, sections were stored overnight in freshly prepared 5x Saline Sodium Citrate (SSC) buffer pH 7.0 (from the 25x: 3M NaCl and 0.3M Sodium Citrate dehydrate in ddH_20). The following day, sections were incubated with the remaining reagents: AMP0 (30 min at 40°C), AMP1 (15 min at 40°C), AMP2 (30 min at 40°C), AMP3 (30 min at 40°C), AMP4 (15 min at 40°C), AMP5 (30 min at RT) and AMP6 (15 min at RT). Slides were rinsed twice with wash buffer between each incubation. A mixture of Fast Red A + B was prepared (1:60) and sections were incubated with the staining solution for 10 min at RT. Gill's hematoxylin (Vector labs) counterstain solution (1:1 in ddH20) was applied, then quickly rinsed and sections were left to dry for 30 min at 60°C. Dried sections were mounted using VectaMount mounting media (Vector labs) and imaged the following day.

Tissue sections were digitised by whole slide-scanning using a Zeiss Axio Scan.Z1 at high resolution (0,11 to 0,15um/px), low to none compression (15% to 0%) and × 40 magnification. When necessary, the whole DG was reconstructed using Adobe Photoshop (Adobe System Incorporated). Each file was then loaded into the open-source software QuPath[64], the DG was manually drawn in each file and the area measured by the software; subsequently the stain was manually counted using the "counter" tool - each red puncta corresponding to a single mRNA molecule. The number of puncta and area were recorded separately, and the density (number of dots per area) in the three GHS-R1a-stained sections was averaged and divided by the density of the positive control stain – in order to account for any difference in tissue quality and RNA preservation. Finally, data were exported to GraphPad Prism (GraphPad Software Inc.) for statistical analysis using one-way ANOVA.

Immunohistochemistry of Human Brain Tissue

Fixed-frozen sections (controls n = 5, PD n = 6, PDD n = 6) stored at −80°C were quickly thawed at RT and fixed for 1 h in 10% NBF (Sigma-Aldrich, UK) to improve adherence to the glass slide. Sections were incubated for 8 min at RT with 1% H_2O_2 (Sigma-Aldrich) in PBST, before incubation for 20 min at RT with 10% normal goat serum (Sigma-Aldrich). This was followed by overnight incubation at 4°C with rabbit anti-GOAT antibody (1:500; Phoenix Pharmaceuticals, Germany). The following day, sections were incubated for 1 h at RT with biotinylated anti-rabbit (1:500; Vector labs, UK) and subsequently for 1 h at RT with pre-mixed avidin-biotin complex (Vector labs, UK). ImmPactDAB (Vector labs, UK) was prepared according to manufacturer's instructions and applied to the sections for 40 s before being washed away with tap water. Nuclei were counterstained for 60 s with Gill's Haematoxylin 1:1 (Vector labs, UK) before the sections were dehydrated and coverslipped using Entellan mounting media (Sigma-Aldrich, UK). Images were acquired by whole slide scanning (as described above). Immunoreactive cells were manually counted within the GCL and the area measured using QuPath software. All analysis was performed in a blinded manner.

Western Blot Assay of Human Brain Tissue

Frozen post-mortem hippocampal tissues were processed for western blot analysis. A total of 18 subjects were included in the western blot analysis. Frozen hippocampal brain tissue (~250mg) was prepared from controls (n = 6), PD (n = 6) and PDD (n = 6). All cases had a post-mortem interval of < 29h. Briefly, protein was extracted using the QIAGEN AllPrep DNA/RNA/Protein mini kit according to manufacturer's instructions (QIAGEN, 80204). Protein was quantified using the Pierce BCA Protein Assay Kit according

to instructions (ThermoFisher, 23227) and the concentration of each sample standardized. Protein samples were combined with 4X sample loading buffer (BioRad, 1610747) and made up to a final volume of 25 μL with water, boiled at 100°C for 5 mins and loaded onto 10% acrylamide gels and separated by SDS-PAGE. The separated proteins were transferred from the gel to the PVDF membrane (BioRad 1620177). The blots were subsequently incubated overnight at 4°C in TBST with 5% BSA with anti-GOAT (1:1000; Phoenix Peptide) and anti-GAPDH (1:5000, Sigma) antibodies. Blots were visualized using the chemiluminescence method (ECL Select, RPN2235; GE Healthcare) and levels were quantified using ImageLab Software v4.1 (ChemiDoc XRS, BioRad) and normalized to GAPDH.

Free Fatty Acid Analysis using Mass Spectrometry

Free fatty acids were extracted from control, PD and PDD human plasma collected at baseline following an overnight fast, using a method as described by Zhang et al.[65]. Briefly 100μl of plasma was added to 250μl of a solvent mixture (1M acetic acid:2-propanol:-hexane (2:20:30)) containing 2.5μg internal standard (nonanoic acid), this was mixed vigorously by vortex followed by the addition of 250μl hexane (ratio of 2.5:1 (solvent mixture:sample)). The samples were mixed vigorously by vortex and centrifuged for 10 minutes at 1100 x g, 4°C. The upper organic layer was removed into clean glass vials. A second extraction step was performed by the addition of 250μl hexane (ratio of 2.5:1, solvent mixture:sample). The organic layers were pooled and dried under N_2 flow or under vacuum, resuspended in Chloroform/Methanol (2:1, v/v) and stored at −70°C. Samples were analyzed by direct infusion electrospray ionisation mass spectrometry (ESI/MS) using the Thermo Scientific LTQ Orbitrap XL (in negative mode). Data was analyzed using Xcalibur software.

QUANTIFICATION AND STATISTICAL ANALYSIS

Statistical analyses were carried out using GraphPad Prizm 6.0 for Mac. Data distribution were assessed using the Schapiro-Wilks normality test. For normally-distributed data, comparison between two groups was assessed by two-tailed unpaired Student's t test. For multiple groups with one variable factor, a one-way ANOVA was used, and where there were two variable factors a two-way ANOVA was used. Appropriate post hoc tests were used as described in the Figure legends. Data displaying non-Gaussian distribution were analyzed by non-parametric tests, as described in the text. Spearman correlation (*two-tailed*) and linear regression analysis were used to determine the goodness-of-fit between plasma AG:UAG (AUC) and cognition (MoCA). Data are presented as mean ± sem. *, $p < 0.05$; **, $p < 0.01$; ***, $p < 0.001$ were considered significant.

Cell Reports Medicine, Volume *1*

Supplemental Information

Unacylated-Ghrelin Impairs Hippocampal Neurogenesis and Memory in Mice and Is Altered in Parkinson's Dementia in Humans

Amanda K.E. Hornsby, Luke Buntwal, Maria Carla Carisi, Vanessa V. Santos, Fionnuala Johnston, Luke D. Roberts, Martina Sassi, Mathieu Mequinion, Romana Stark, Alex Reichenbach, Sarah H. Lockie, Mario Siervo, Owain Howell, Alwena H. Morgan, Timothy Wells, Zane B. Andrews, David J. Burn, and Jeffrey S. Davies

Figure S1. Hornsby *et al*.

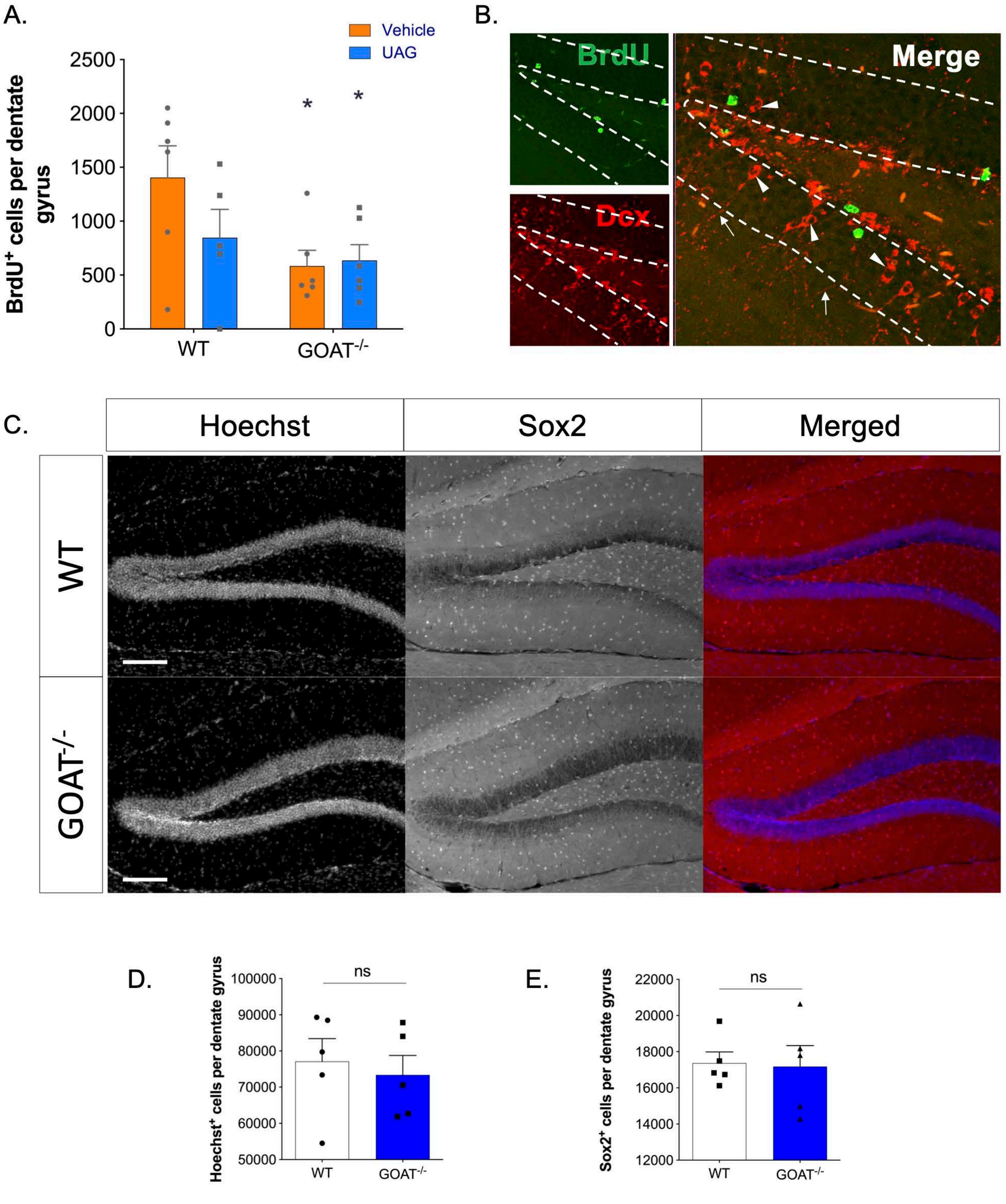

Figure S1. GOAT$^{-/-}$ mice display reduced new BrdU^{+} cell number in the DG compared to WT mice (A). Representative confocal image showing BrdU (green) and Dcx (red) in the wild-type mouse hippocampus. Arrowheads show Dcx^{+} cell bodies within the GCL, whilst arrows depict Dcx^{+} projections extending into the molecular layer (B). Statistical analysis was performed by 2-way ANOVA followed by Holm-Sidak *post-hoc* comparisons vs WT vehicle group (*n*=5-6 mice/group). Fluorescent images of Hoechst^{+} nuclei and Sox2^{+} NSPCs in DG from WT and GOAT$^{-/-}$ mice (C). GOAT$^{-/-}$ mice do not have gross anatomical changes (Hoechst^{+})(D) or impaired NSPC (Sox2^{+})(E) cell number in the granule cell layer of the DG. Statistical analysis performed by Student's *t*-test (*n*=5 mice/group). *P<0.05. Scale bar = 200µm. All data shown are mean ± SEM. Related to Figures 1 - 4.

Figure S2. Hornsby *et al.*

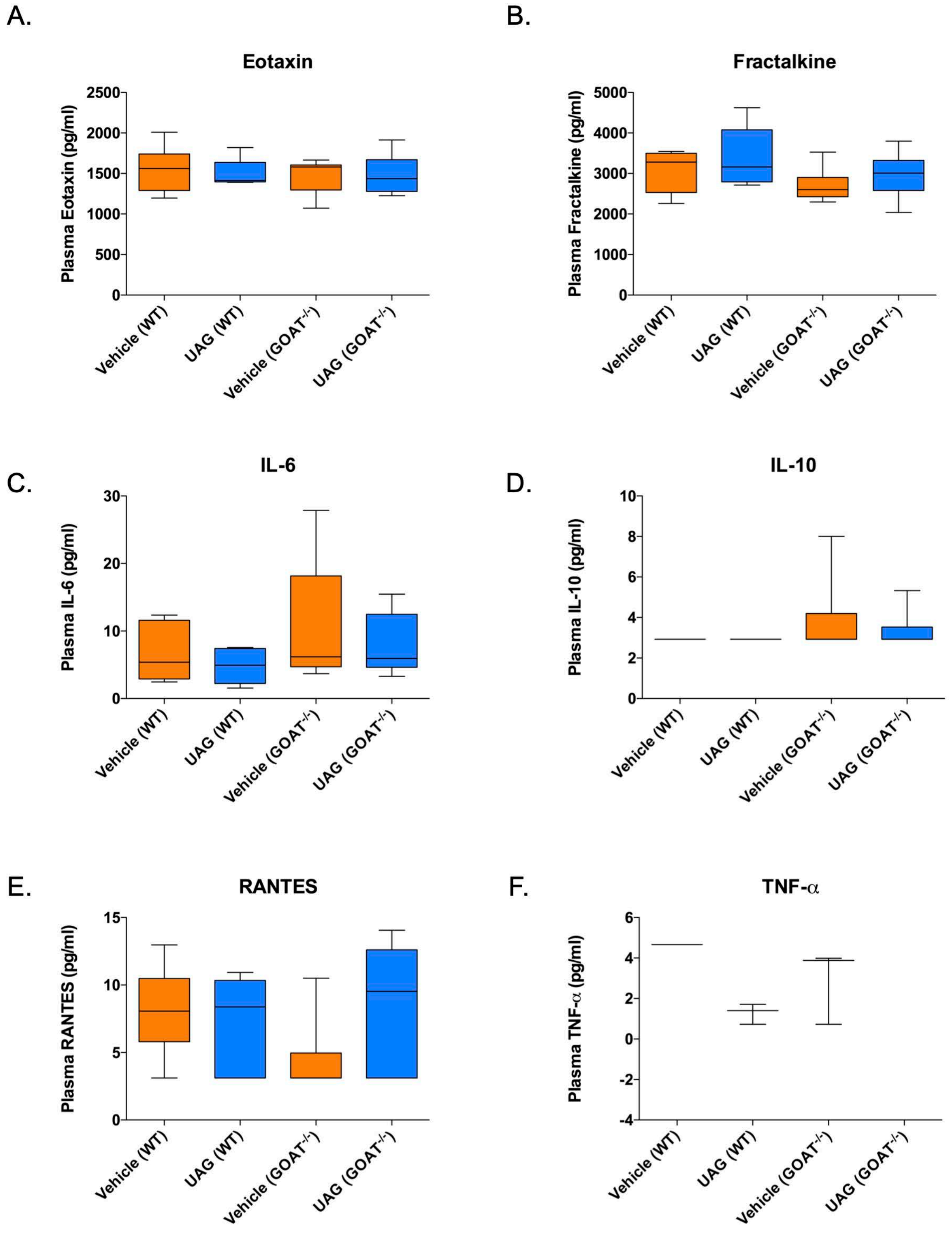

Figure S2. Peripheral administration of unacylated-ghrelin (UAG) or genetic ablation of GOAT does not affect quantities of circulating factors that are known to modulate neurogenesis (A-F). Statistical analysis was performed by 2-way ANOVA followed by Holm-Sidak *post-hoc* comparisons vs WT vehicle group. *P<0.05. *n* = 5-6 mice/group. All data shown are mean ± SEM. Related to Figures 1 – 4.

Figure S3. Hornsby *et al.*

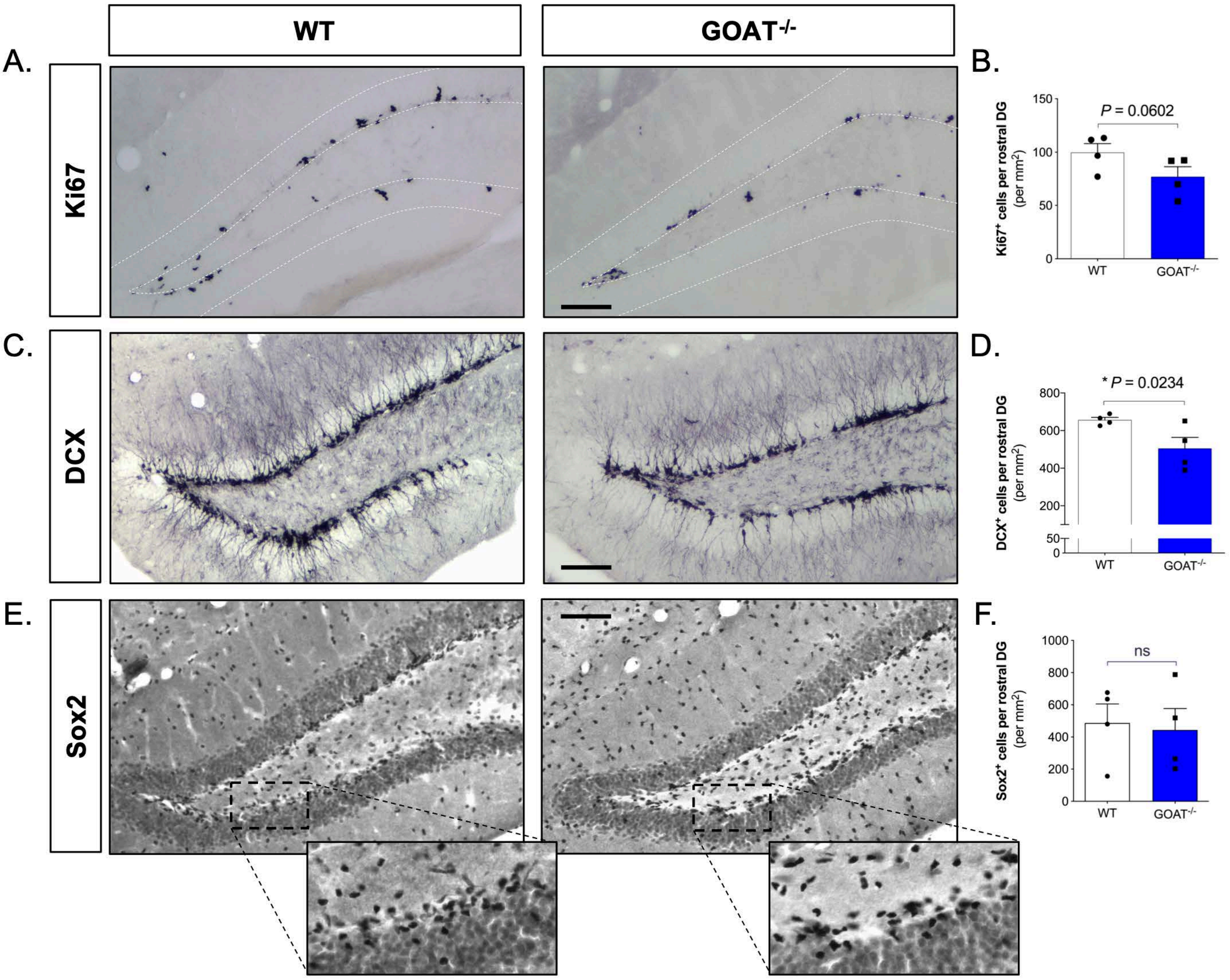

Figure S3. GOAT$^{-/-}$ mice[37] have a reduced rate of cell proliferation (A,B) and neurogenesis (C,D) in the hippocampal DG. However, NSPC (Sox2^{+}) number in the SGZ of the hippocampal DG is similar between WT and GOAT$^{-/-}$ mice (E,F). Brains were collected at 12-weeks of age following intra-cardial perfusion with 4% PFA prior to processing for IHC. Statistical analysis was performed by Student's *t*-test. Scale bar = 200μm. *n* = 4 mice/group. All data shown are mean ± SEM. *P<0.05. Related to Figures 1 and 2.

Figure S4. Hornsby *et al.*

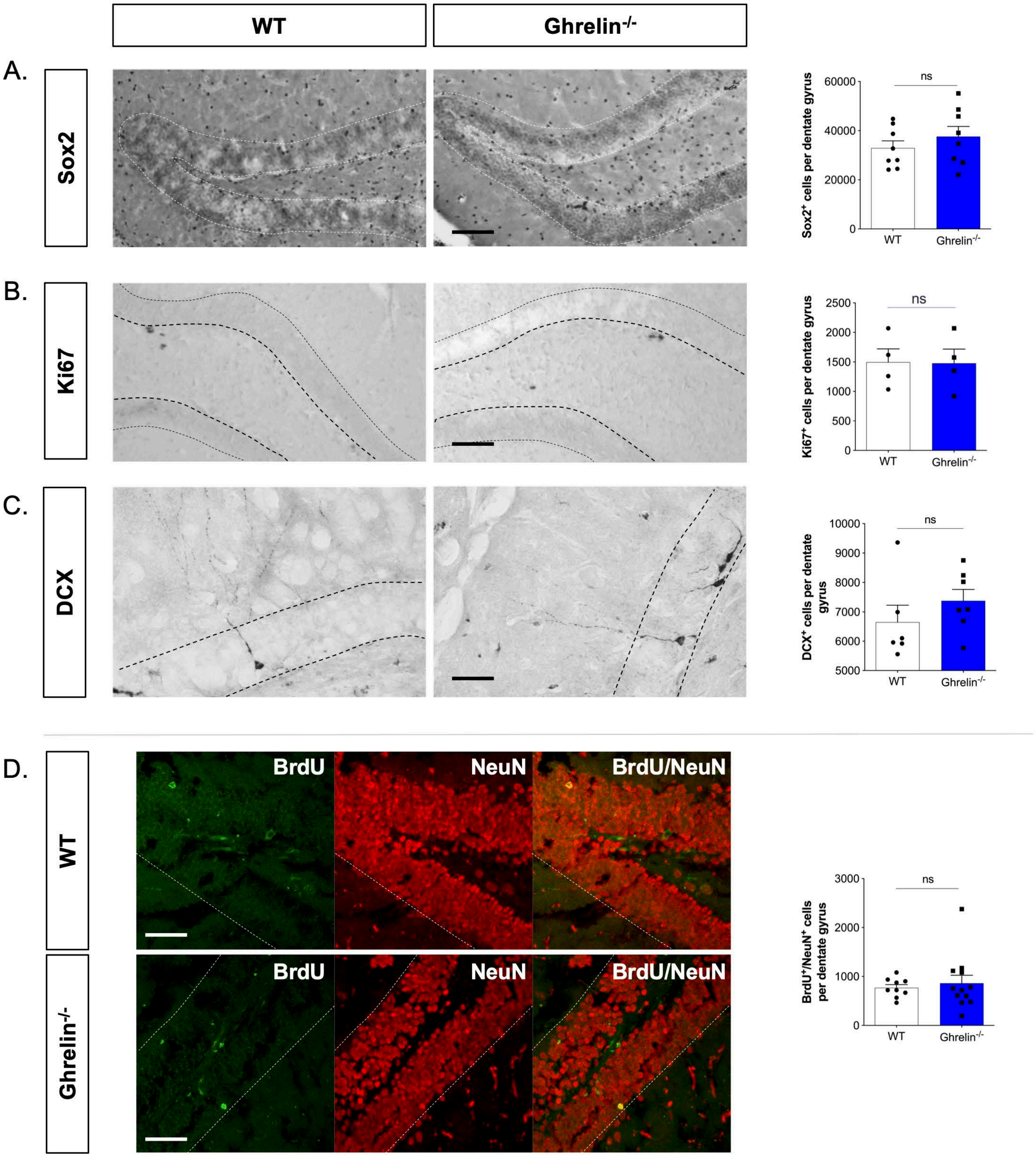

Figure S4. Ghrelin$^{-/-}$ mice have unaltered numbers of Sox2^{+} neural stem cells (n=8/group) (A), Ki67^{+} dividing NSPCs (WT n=4/group)(B), Dcx^{+} immature neurons (WT n=6, ghrelin$^{-/-}$ n=7)(C) and BrdU^{+}/NeuN^{+} new adult born neurons (WT n=9, ghrelin$^{-/-}$ n=12) (D) in the hippocampal DG. Statistical analysis was performed by Students t-test. Scale bar = 200µm for A&B, 50µm for C&D. ns = not significant. All data shown are mean ± SEM. Related to Figures 1 and 2.

Figure S5. Hornsby *et al.*

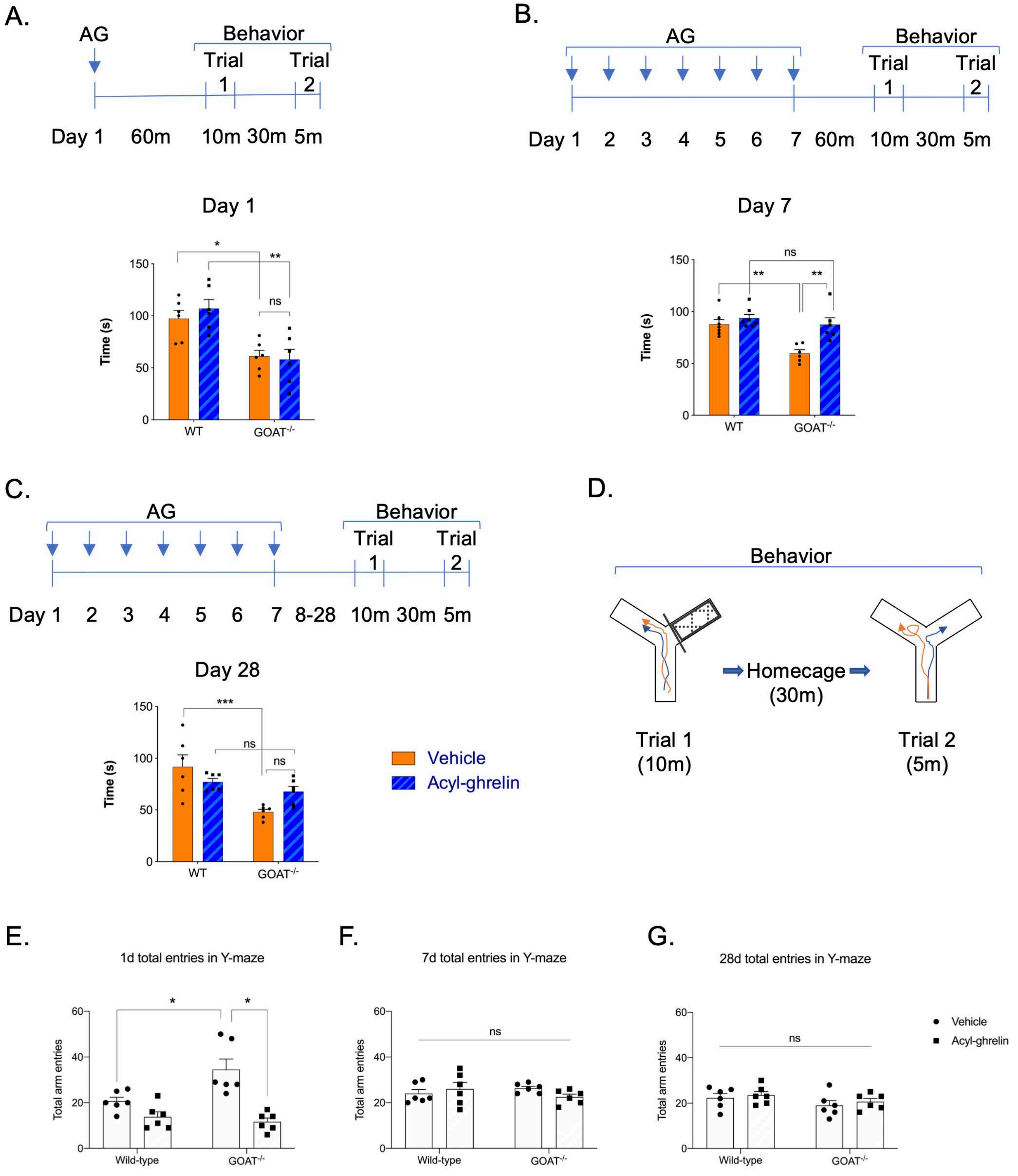

Figure S5. Adult GOAT$^{-/-}$ mice display hippocampal-dependent spatial memory deficits that are rescued by acyl-ghrelin treatment. GOAT$^{-/-}$ mice display memory impairments (A) that are restored by acyl-ghrelin treatment for 7 days (B) and 21 days following the end of treatment on day 28 (C). Schematic representations of treatment timelines (A-C) and behavior using Y-maze (D) are shown. Vehicle-treated GOAT$^{-/-}$ mice display increased total arm entries relative to vehicle-treated wild-type mice that are normalized by acyl-ghrelin treatment on day 1 (E) but show no changes on days 7 and 28 (F,G). Statistical analysis was performed by 2-way ANOVA followed by Holm-Sidak *post-hoc* comparisons (*n*=6 mice/group). *P<0.05, **P<0.01, ***P<0.001. All data shown are mean ± SEM. Related to Figure 4.

Figure S6. Hornsby *et al.*

A.

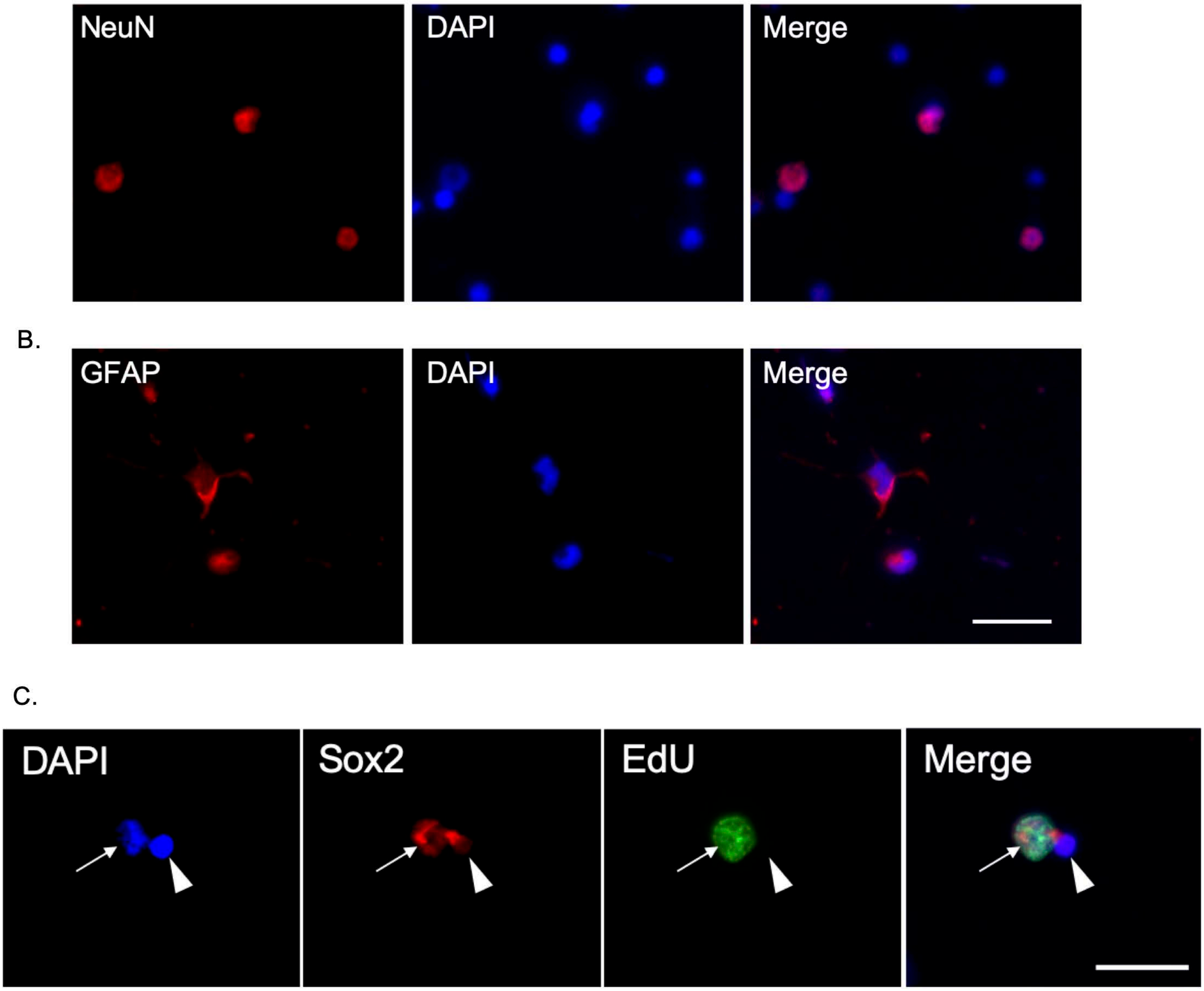

Figure S6. Primary rat hippocampal cell cultures contain a mix of $NeuN^+$ neurons (A) and astrocytes (B) to determine the indirect direct effects of acyl-ghrelin on neurogenesis. These cells also contain dividing (EdU^+) NSPCs ($Sox2^+$) identified by the white arrow; whilst a non-dividing NSPC is identified by the white arrow-head (C). Scale bar = 20**µ**m). Related to Figures 5 and 6.

Figure S7. Hornsby *et al.*

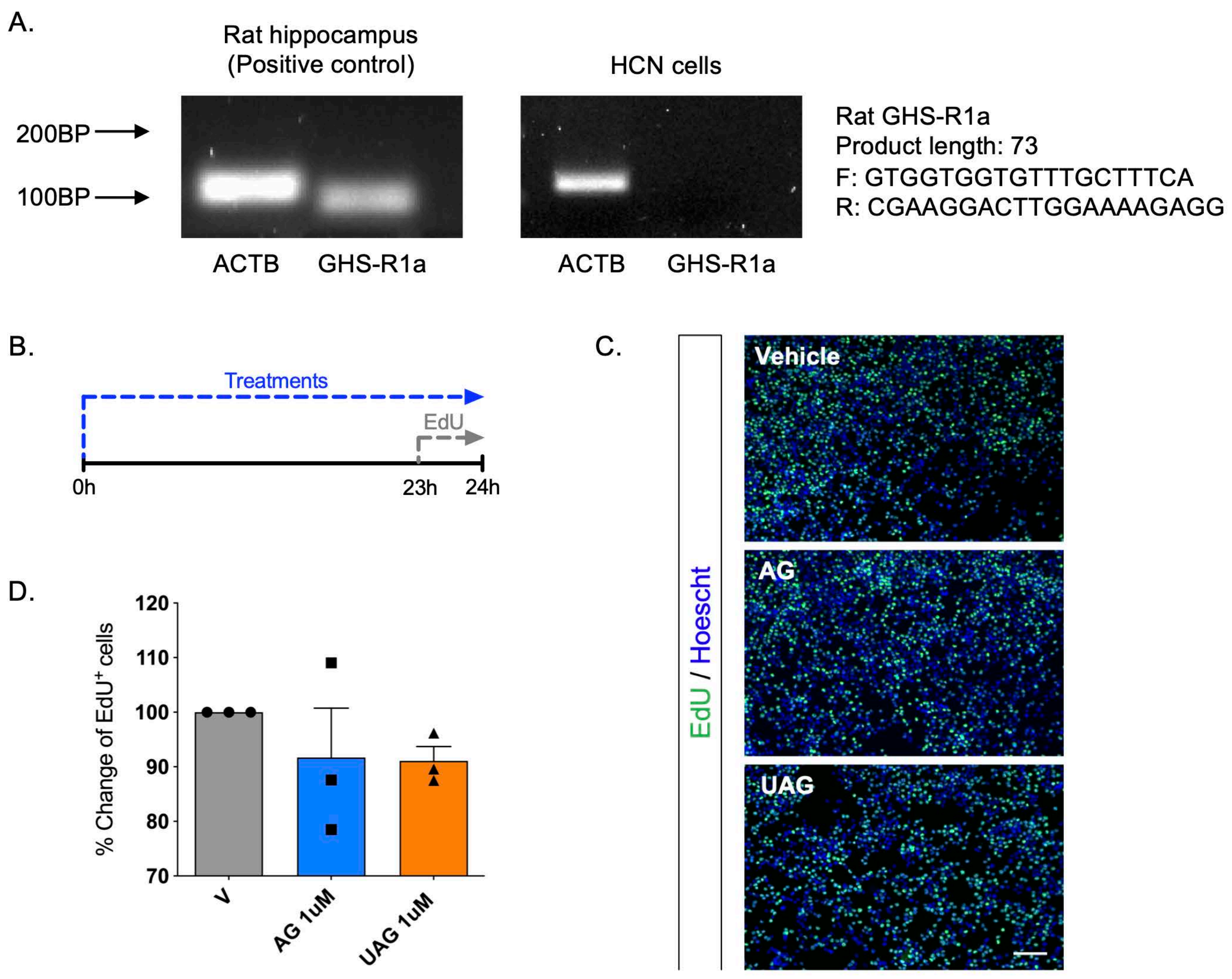

Figure S7. Hippocampal Neural Stem/Progenitor Cells (NSPCs) do not express GHS-R1a. PCR assays reveal that adult rat hippocampal homogenate express GHS-R1a, alongside the housekeeping gene, ACTB. However, rat-derived hippocampal NSPCs (HCN cells), do not express GHS-R1a (A). Schematic of *in-vitro* experiment to determine direct effects on NSPC proliferation (B). Acyl-ghrelin and unacylated-ghrelin do not stimulate cell proliferation (EdU^+) in NSPCs (C, D). Scale bar = 200**µ**m. Related to Figure 6.

Figure S7. Hornsby *et al.*

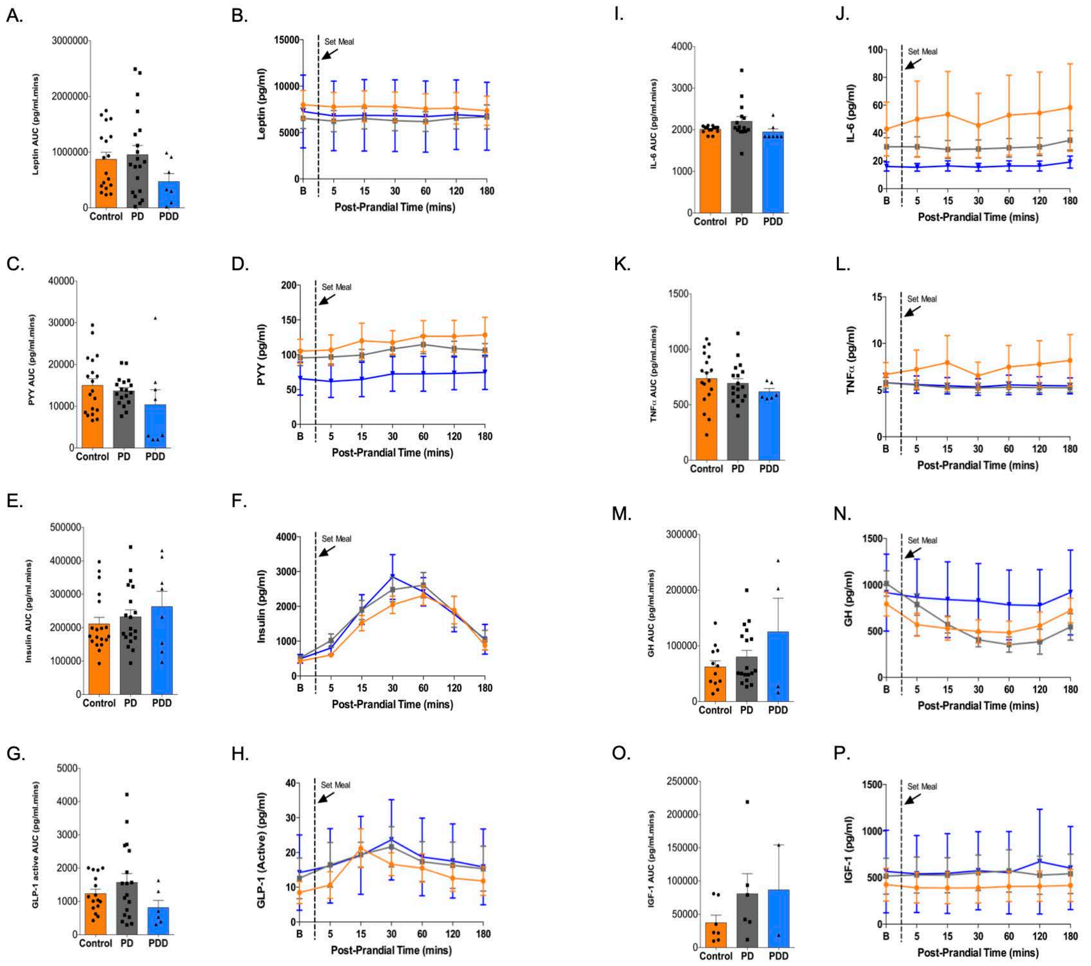

Figure S8. Quantification of circulating factors in healthy, PD and PD dementia subjects. Plasma analysis in healthy controls (n=20), PD (n=20) and PDD (n=8) subjects under fasting and post-prandial conditions. Area under curve (AUC) and plasma values across 180 min demonstrate no change in leptin (A,B), PYY (C,D), insulin (E,F), GLP-1 (active) (G,H), IL-6 (I,J), TNF**α** (K,L), GH (M,N) and IGF-1 (O,P). Dotted line indicates breakfast consumption at time 0. Statistical analysis performed by Kruskal-Wallis test with Dunn's post-hoc multiple comparison revealed no differences between groups. All data shown are mean ± SEM. Related to Figure 7.

Table S1. Hornsby *et al.*

	Healthy controls (*n*=20)	Parkinson's disease (n=20)	Parkinson's disease dementia (*n*=8)	Significance
Age (mean ± sd)	74 ± 6.28	72.2±5.51	74.75 ± 5.99	*P* =0.510
Male (%)	55%	55%	87.5%	*P* =0.229+
MoCA (mean ± sd)	28.35 ± 1.14	27.55 ± 1.47	14.12 ± 5.14	*P* = 0.000*
BMI (mean ± sd)	25.75 ± 2.04	25.31±2.82	24.41 ± 3.20	*P* =0.532
Fasted total ghrelin (pg/ml) (mean ± sem)	541.0±87.98	647.9±108.7	377.8±77.00	*P* =0.2896
Fasted acyl-ghrelin (pg/ml) (mean ± sem)	78.93±13.77	114.4±18.82	51.39±17.24	*P* =0.0925
Post-prandial (180 min) total ghrelin (pg/ml) (mean ± sem)	500.4 ± 65.80	566.1 ± 96.89	401.8 ± 88.61	*P* =0.6464
Post-prandial (180 min) acyl-ghrelin (pg/ml) (mean ± sem)	72.86 ± 10.75	112.0 ± 18.31	41.32 ± 18.25	*P* =0.0169*

Table S1. Demographic information for study participants. MoCA; Montreal Cognitive Assessment. BMI; Body Mass Index. Statistical analysis was performed by Kruskal-Wallis test or the Chi-squared test+. *P <0.05 considered significant. Related to Figure 7.
